# Factors associated with antiretroviral therapy interruption in hospitalized people living with HIV: a retrospective cohort*

**DOI:** 10.1590/1980-220X-REEUSP-2025-0359en

**Published:** 2026-05-22

**Authors:** Jarbas da Silva Ziani, Laís Mara Caetano da Silva Corcini, Jenifer Härter, Caren Fabiana Alves, Stela Maris de Mello Padoin, Cláudia Zamberlan, Gabriela Pozzobon Zamberlan da Silva, Francielle Liz Monteiro

**Affiliations:** 1 Universidade Federal de Santa Maria, Centro de Ciências da Saúde, Santa Maria, RS, Brazil.; 2 Universidade Federal do Pampa, Departamento de Enfermagem, Uruguaiana, RS, Brazil.; 3 Universidade Franciscana, Departamento de Enfermagem, Santa Maria, RS, Brazil.; 4 Universidade Franciscana, Departamento de Medicina, Santa Maria, RS, Brazil.; 5 Universidade Franciscana, Departamento de Biomedicina, Santa Maria, RS, Brazil.

**Keywords:** HIV, Antiretroviral Therapy, Highly Active, Hospitalization, Medication Adherence, Risk Factors

## Abstract

**Objective:**

To identify the factors associated with the interruption of antiretroviral therapy (ART) in people living with HIV hospitalized in a medium-sized hospital in Rio Grande do Sul.

**Method:**

Retrospective and documentary cohort study with 208 participants, using data from electronic medical records from 2022 to 2024. The outcome was the discontinuation of ART, analyzed by sociodemographic and clinical characteristics, and therapeutic history. Chi-square, Fisher’s exact test, and Poisson regression with robust variance (p < 0.05) were applied.

**Results:**

The prevalence of discontinuation was 39.4% (95% CI: 33.0–45.8). The highest risk of interruption was observed among men (PR = 1.58; 95% CI: 1.22–2.04), alcohol users (PR = 1.46; 95% CI: 1.10–1.94), users of other drugs (PR = 1.98; 95% CI: 1.45–2.72), homeless people (PR = 1.38; 95% CI: 1.08–1.76), those with a travel time to the unit greater than 31 minutes (PR = 1.67; 95% CI: 1.29–2.16), and those with a previous history of interruption (PR = 1.52; 95% CI: 1.16–1.99).

**Conclusion:**

Interruption of ART is related to social and behavioral vulnerabilities, requiring intersectoral actions and nursing practices focused on continuity of care and strengthening of public policies to combat HIV.

## INTRODUCTION

Infection with the Human Immunodeficiency Virus (HIV) and acquired immunodeficiency syndrome (AIDS) remain major global public health problems. By 2023, more than 42.3 million people had died as a result of AIDS, with approximately 630,000 deaths in that year alone, largely due to opportunistic infections associated with non-adherence to antiretroviral therapy (ART)^(1)^.

To address this scenario, the Joint United Nations Programme on HIV/AIDS (UNAIDS) proposed the “95-95-95” target, aligned with Sustainable Development Goal (SDG) No. 3, which seeks to eliminate the epidemic by 2030^(1)^. Despite the progress made, Brazil has not yet fully achieved these goals, having only reached the third milestone, related to viral suppression^(2)^.

Between 2007 and 2023, the country registered 489,594 cases of HIV, of which 19.1% occurred in the South region and 42,456 in Rio Grande do Sul^(2)^. This state has historically maintained high incidence and mortality rates, highlighting the need to strengthen the healthcare network, especially in the coordination between Primary Health Care (PHC) and Specialized Care Services (*SAE*), which are fundamental for longitudinal monitoring and continuous therapeutic support.

ART is the main therapeutic resource for controlling HIV infection, reducing morbidity and mortality, preventing hospitalizations, and interrupting the chain of transmission through the concept of “Undetectable = Untransmittable (U = U)”^(3)^. However, treatment interruption or irregular adherence still represents a significant obstacle, influenced by social vulnerabilities, access barriers, stigma, and the use of psychoactive substances^(4,5,6)^.

Most studies on adherence to ART focus on people followed up on an outpatient basis, with few investigations addressing the reality of hospitalized patients, a group that presents greater clinical severity and faces additional challenges in the continuity of care^(4,7,8)^.

Therefore, this study aims to identify the factors associated with the interruption of antiretroviral therapy in people living with HIV hospitalized in a medium-sized hospital in Rio Grande do Sul, contributing to the improvement of care practices and public policies aimed at maintaining treatment and integrating levels of care.

## METHOD

### Study Design, Period and Local

This quantitative, retrospective cohort study analyzed electronic medical records of hospitalized HIV-positive individuals admitted between January 1, 2022, and January 31, 2024, and followed from admission to hospital discharge or death, investigating the interruption of ART as an outcome. The methodological description of this research was directed by the guidelines of *Strengthening the Reporting of Observational Studies in Epidemiology* (STROBE).

The research was conducted in a clinical inpatient unit of a teaching hospital located in the state of Rio Grande do Sul, Brazil. The setting was chosen because the institution is a benchmark in the care of people with infectious diseases. The institution has a public and philanthropic nature, serving as a reference for 33 municipalities in the central-western macro-region of Rio Grande do Sul. This macro-region encompasses an estimated population of over 500,000 inhabitants. Regarding the study’s development unit, it consists of 34 beds, with 15 for men, 15 for women, and four isolation beds.

### Population, Inclusion and Exclusion Criteria

The population consisted of people diagnosed with HIV who were admitted to the inpatient unit between January 1, 2022, and January 31, 2024. This period was established because, during the years 2020 to 2021, the unit became a reference point for the care of people with COVID-19. In this way, the aim was to minimize the effects of the pandemic on the data obtained and analyzed.

The inclusion criteria were: electronic medical records of people over 18 years of age, diagnosed with HIV and hospitalized for at least 72 hours. This timeframe was considered aiming at obtaining the minimum information necessary for the study. Follow-up was conducted from hospital admission until discharge or death. The exclusion criteria were: medical records that could not be located or that did not contain sufficient information; medical records in which the HIV diagnosis was ruled out or remained inconclusive during hospitalization; and individuals who started ART during hospitalization, since the study focuses on treatment interruption before this event. No data were collected from individuals who were readmitted after the study period.

A minimum sample of 188 medical records was considered, calculated based on the total number of HIV hospitalizations in the unit over the last five years, adopting a 5% error tolerance, a 95% confidence level, and a proportion of the characteristic of interest of 0.5 (50%), a conservative value chosen because the exact prevalence of therapy interruptions in this hospital population was unknown.

### Data Collection

Data collection took place between August 2023 and April 2024, conducted by the author of the research and a volunteer data collector, both previously trained to minimize any data collection biases.

**Figure 1:**
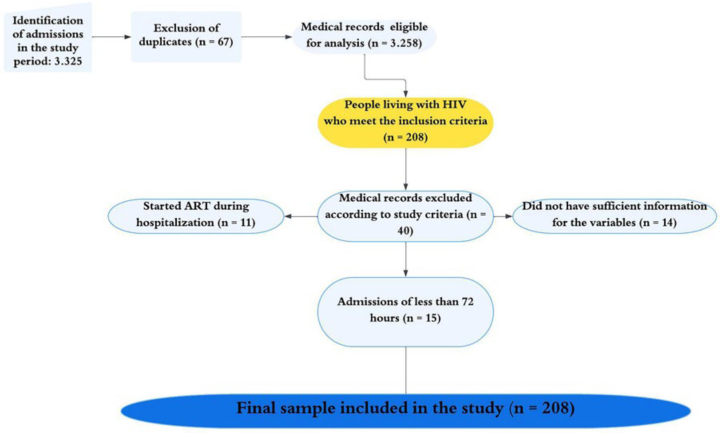
Flowchart of the number of people eligible for the study (n = 208) – Santa Maria, RS, Brazil, 2022–2024.

Source: Prepared by the author (2025).

To collect the data, a search was conducted in the institution’s electronic medical records database, including all individuals admitted to the inpatient unit during the study period. A total of 3,325 hospitalizations were identified, and after excluding duplicates, 3,258 medical records were included. Subsequently, the medical records of people living with HIV were identified. For this stage, the medical records were analyzed in full to select those that met the study’s inclusion criteria, totaling 248 records of people living with HIV. After applying the exclusion criteria, a final sample of 208 participants was obtained, of which 40 records were excluded from the study (15 because they belonged to hospitalizations lasting less than 72 hours, 14 because they did not present sufficient information for the variables studied, and 11 because they started using ART during hospitalization). The detailed sample flow is also presented visually in Figure 1.

### Data and Variable Collection Instrument

The instrument was developed based on the recommendations of the Clinical Protocol and Therapeutic Guidelines for the Management of HIV Infection in Adults from the Ministry of Health (MS) of 2018 and consisted of the sociodemographic variables sex (male/female), skin color (white and black/brown), education (less than eight years of study and more than eight years of study), and economic class. The economic class stratification was carried out according to the classification already used by the institution where the study was conducted, which categorizes the monthly family income of the participants as follows: Class A: monthly income greater than R$ 11,262; Class B: between R$ 8,641 and R$ 11,261; Class C: between R$ 2,005 and R$ 8,640; Class D: between R$ 1,255 and R$ 2,004; Class E: between R$ 0.00 and R$ 1,254. Sexual orientation (homosexual, heterosexual, and bisexual), marital status (single and married), and fixed relationship (yes or no) were also included. Regarding social and behavioral vulnerability factors, the following dichotomous variables (yes or no) were included: tobacco-related disorder, alcohol-related disorder, homelessness, drug and/or alcohol use, and deprivation of liberty.

The clinical variable was AIDS (yes or no). For the classification of this variable, the criteria of the Ministry of Health were adopted. The case was considered as AIDS when there was an explicit record of the diagnosis in the medical record or when it met laboratory criteria (CD4+ T cells < 200 cells/mm^3^ or viral load >1,000 copies/mL^(3)^) and/or compatible clinical signs (worsening of the condition associated with opportunistic infections). In the absence of laboratory data, clinical criteria and/or ICD code registration prevailed to reduce cases loss.

The type of hospital admission (elective or urgent), coinfection (tuberculosis/HIV), and viral load (undetectable, low, and high) were also considered. Viral load was categorized as: undetectable, up to 200 copies/mL (no risk of sexual transmission); low, between 200 and 1,000 copies/mL (almost negligible risk of sexual transmission); high, greater than 1,000 copies/mL (significant risk of sexual transmission)^(2)^; and CD4+ T cells (CD4+ T cells greater than 201 cells/mm^3^ and CD4+ T cells lower than 200 cells/mm^3^). In addition, the history of previous hospitalizations (yes or no) and the outcome of the hospitalization (hospital discharge or death) were considered. It should be noted that the instrument was shared with a committee of five experts in the field of HIV and epidemiology, and was evaluated and validated in terms of its content and semantics through the calculation of the Content Validity Index (CVI), with the aim of improving it. Items with a CVI lower than 0.80 were reviewed or removed.

The sample was stratified into two groups, considering the variable of treatment interruption, which was analyzed based on the time criterion. The MS defines interruption as the absence of medication pickup for a period exceeding 28 days after the scheduled date for the last dispensing, associated with the absence from outpatient follow-up^(3)^. It should be noted that this variable was available in the patient’s medical record, as the municipality’s *SAE* shares this information with the hospital.

Regarding the therapeutic history, the following variables were considered: history of previous interruption of ART (yes or no), number of ART regimens (first, second and third or more regimens), medication pick-up unit (outpatient or Specialized Care Service/Testing and Counseling Center - *SAE/CTA*), reason for seeking diagnosis (opportunistic infections, symptomatology or rapid testing) and travel time to the medication pick-up unit (less than 30 minutes and more than 31 minutes).

To structure the variable “travel time to the medication pick-up unit,” the person’s home address and reference unit were used. To estimate the time, the variable was measured using the tool *Google Maps*, taking into account the journey by public transport. Specifically, the estimated average travel time under normal traffic conditions was used, avoiding the fluctuation of actual traffic, which is difficult to standardize retrospectively.

The stratification of travel time (≤30 minutes and ≥31 minutes) was defined considering the geographical and mobility characteristics of the municipality, in which journeys exceeding 30 minutes represent an access barrier frequently observed in urban routes. Thus, the cutoff point reflects both evidence from the literature on geographical barriers to access to health services and the local reality of urban displacement^(4,7)^.

### Data Analysis

A spreadsheet database was created in *Excel® (Microsoft, version 10, USA)*, with double data entry, followed by validation to minimize measurement bias and ensure data reliability. After validating the spreadsheet and making corrections, the data was transferred to a final database, which was then imported for analysis in the software *Statistical Package for the Social Sciences* (SPSS), version 20.0.

For data analysis, descriptive statistics were used to characterize the sample. For categorical variables, absolute (n) and relative (%) frequencies were presented. For the numerical variables, the normality of the distribution was verified using the Kolmogorov-Smirnov test. Variables with a normal distribution were described by the mean and standard deviation (SD±), while variables with an asymmetrical distribution were presented by the median and the interquartile range.

The association between categorical variables was assessed using Pearson’s Chi-Square test and Fisher’s Exact Test, when appropriate, i.e., when the Chi-Square assumptions were not met, such as cells with expected counts less than five, for example. A statistical significance level of 5% (p < 0.05) was adopted.

A multivariate analysis was also conducted using Poisson regression with robust variance, with the aim of identifying factors associated with the interruption of antiretroviral therapy. Before constructing the models, multicollinearity among the predictor variables was verified using the Variance Inflation Factor (VIF), with acceptable values below 5. The variables were included in successive thematic blocks: sociodemographic (Block 1), social and behavioral vulnerabilities (Block 2), and clinical/therapeutic history (Block 3). Variables with p-values < 0.20 in the bivariate analysis were considered for inclusion in the initial model of each block, a criterion recommended by the scientific literature to maximize the chance of including relevant variables in the multivariate model^(9)^. The permanence in the final model was determined based on statistical significance (p < 0.05) and epidemiological relevance.

The conceptual model of this study posits that discontinuation of ART is influenced by social and behavioral vulnerabilities, and clinical/therapeutic history. The primary outcome, discontinuation of ART, was defined according to the criteria of the *MS*. For the construction of the causal model, potential confounding variables, widely evidenced in the literature as influencing the outcome of ART discontinuation, were considered, including viral load, CD4+ T cell count, AIDS diagnosis, hospitalization outcome, type of hospital admission, previous discontinuation, and tuberculosis-HIV coinfection.^(1,3,4,7,8)^. The impact of these confounding variables was adjusted in the multivariate analyses to better estimate the association between the factors of interest and the interruption of ART.

This study has some limitations inherent to its retrospective design. First, reliance on medical records can lead to information bias (underreporting), since the quality and completeness of the data depend on the clinical record. Secondly, the study was conducted in a reference hospital that concentrates on more severe cases, which may generate selection bias and limit the generalizability of the results to other populations of people with HIV. Finally, the use of secondary data prevents the definitive establishment of causality.

### Ethical Aspects

The study was conducted in accordance with national and international ethical guidelines and was approved by the Research Ethics Committee of the Universidade Franciscana with opinion number 5.828.312. The Free Informed Consent Form was waived, since the data was obtained through electronic medical records.

## RESULTS

A total of 248 medical records of people living with HIV who were hospitalized during the study period were eligible. Of these, 40 were excluded (15 due to hospitalizations of less than 72 hours, 14 due to insufficient information, and 11 due to initiation of ART during hospitalization). Thus, the final sample consisted of 208 medical records, with 82 (39.4%) showing interruption of ART and 126 (60.6%) without interruption.

The mean age of the participants was 39.6 years (SD ± 13.2; range 18–89), and 82 (39.4%) had discontinued their ART. The mean interruption time was 14.2 months (SD ± 8.0; median 12; range 5–47 months).

In the bivariate analysis, a significant association was observed between discontinuation of ART and male sex, black/brown skin color, and lower education level (< 8 years), economic class E, individual income and absence of a fixed partnership (p < 0.001), as per Table 1. The other sociodemographic variables did not show a statistically significant association.

**Table 1:** Sociodemographic characteristics of people living with HIV who are hospitalized, according to ART interruption (n = 208) – Santa Maria, RS, Brazil, 2022–2024.

Variables	Category	Interruption		No interruption		Total		p-value^* †^
		n	%	n	%	n	%	
Sex								< 0.001^†^
	Male	66	80.5	58	46.0	124	59.6	
	Female	16	19.5	68	54.0	84	40.4	
Self-declared race								< 0.001^†^
	Black/brown	67	81.7	52	41.3	119	57.2	
	White	15	18.3	74	58.7	89	42.8	
Educational level								< 0.001^†^
	0–8	66	80.5	27	21.4	93	44.7
	≥ 9	16	19.5	99	78.6	115	55.3	
Economy class^§^								< 0.001^‡^
	Class C	1	1.9	52	98.1	53	25.5	
	Class D	14	22.6	48	77.4	62	29.8	
	Class E	67	72.0	26	28.0	93	44.7	
Monthly income								< 0.001^†^
	Individual	72	87.8	41	32.5	113	54.3	
	Family	10	12.2	85	67.5	95	45.7	
Sexual orientation								0.441^†^
	Homosexual	27	45.8	32	54.2	59	28.4	
	Heterosexual	46	36.6	87	65.4	113	63.9	
	Bisexual	9	56.2	7	43.8	16	7.7	
Marital status								< 0.001^†^
	Single	74	90.2	36	28.6	110	52.9	
	Married	8	9.8	90	71.4	98	47.1	
Fixed Relationship								< 0.001^‡^
	Yes	4	4.9	90	71.4	94	45.2	
	No	78	95.1	36	28.6	114	54.8	

Legend: *p-value = Significance level (p < 0.05);

^†^ = Pearson’s chi-square test;

^‡^ = Fisher’s exact test;

^§^ = Economic class stratification: performed according to the classification used by the institution: Class A = monthly income > R$ 11,262; Class B = R$ 8,641–11,261; Class C = R$ 2,005–8,640; Class D = R$ 1,255–2,004; Class E = R$ 0–1,254. No participant was classified in classes A or B.

Interruption of ART showed a statistically significant association with social, behavioral, and clinical vulnerability factors. Among the behavioral variables, a higher prevalence of cessation was observed among people who used tobacco (86.6%), consumed alcohol (87.8%), used other drugs (74.4%), and lived on the streets (42.7%) (p < 0.001). No significant association was identified with the variable deprivation of liberty (p = 0.378).

Among clinical factors, discontinuation of ART was associated with a diagnosis of AIDS (86.6%), high viral load (95.1%), and CD4+ T-cell count below 200 cells/mm^3^ (89.0%) (p < 0.001), urgent hospitalizations (81.7%). A history of previous hospitalizations also showed a significant association, being observed in 79.3% of those who interrupted treatment (p < 0.001). Regarding the outcome of hospitalization, interruption was more prevalent among patients who died (65.9%) compared to those who were discharged, a statistically significant difference (p < 0.001). Tuberculosis/HIV coinfection did not show a significant association with ART interruption (p = 0.092), as shown in Table 2.

**Table 2:** Vulnerability factors and clinical conditions associated with ART interruption in hospitalized people living with HIV (n = 208) – Santa Maria, RS, Brazil, 2022–2024.

Variables	Category	Interruption		No interruption		Total		p value^*^
		n	%	n	%	n	%	
Block 1 – Vulnerability Factors								< 0.001^†^
Tobacco use	Uses	71	86.6	40	31.7	111	53.4	
	Does not use	11	13.4	86	68.3	97	46.6	
Alcohol consumption								< 0.001^†^
	Uses	72	87.8	30	23.8	102	49.0	
	Does not use	10	12.2	96	76.2	106	51.0	
Use of other drugs								< 0.001^†^
	Uses	61	74.4	13	10.3	74	35.6	
	Does not use	21	25.6	113	89.7	134	64.4	
Homelessness								< 0.001^†^
	Yes	35	42.7	10	7.9	45	21.6	
	No	47	57.3	116	92.1	163	78.4	
Deprived of liberty								
	Yes	5	6.1	12	9.5	17	8.2	0.378^‡^
	No	77	93.9	114	90.5	191	91.8	
Block 2 – Clinical conditions								
Diagnosis of AIDS^§^								< 0.001^†^
	Yes	71	86.6	27	21.4	98	47.1	
	No	11	13.4	99	78.6	110	52.9	
HIV/Tuberculosis coinfection								0.092^†^
	Yes	61	74.4	20	15.9	81	38.9	
	No							
Block 3 – Laboratory tests								
Viral Load^||^								< 0.001^‡^
	High	78	95.1	53	42.1	131	63.0	
	Low	3	3.7	37	29.4	40	19.2	
	Undetectable	1	1.2	36	28.6	37	17.8	
CD4+^¶^ T cells								< 0.001^†^
	< 200	73	89.0	52	41.3	125	60.1	
	> 201	9	11.0	74	58.7	83	39.9	
Block 4 – History and outcome of hospitalization								
Nature of the hospitalization								< 0.001^†^
	Urgent	65	81.7	40	31.7	107	51.4	
	Elective	15	18.3	86	68.3	101	48.6	
History of previous hospitalizations								< 0.001^†^
	Yes	65	79.3	37	29.4	102	49.0	
	No	17	20.7	89	70.6	106	51.0	
Outcome of hospitalization								< 0.001^†^
	Hospital discharge	28	34.1	106	84.1	134	64.4	
	Death	54	65.9	20	15.9	74	35.6	

Legend: *p-value = Significance level (p < 0.05);

^†^Pearson’s chi-square test;

^‡^Fisher’s Exact Test;

^§^ = The diagnosis of AIDS was defined according to the criteria of the MS: presence of opportunistic infections, viral load >1,000 copies/mL, CD4+ T cells < 200 cells/mm^3^ or record with corresponding ICD code;

^||^ = Viral load was categorized as: undetectable (≤ 200 copies/mL), low (200–1,000 copies/mL), and high (> 1,000 copies/mL);

^¶^CD4+ T lymphocytes were considered low when ≤ 200 cells/mm^3^ and high when > 200 cells/mm^3^.

Regarding therapeutic history, discontinuation of ART showed a statistical association with the number of ART regimens (with a higher frequency of discontinuation in the third or greater number of regimens completed), and the medication dispensing unit (*SAE/CTA*) (Table 3). Additionally, interruptions were more prevalent among people who sought HIV diagnosis due to opportunistic infections or symptoms, and those who took more than 31 minutes to travel to the medication pick-up unit.

**Table 3:** Therapeutic history among people living with HIV in hospital, according to ART interruption (n = 208) – Santa Maria, RS, Brazil, 2022–2024.

Variables	Category	Interruption		No interruption		Total		p value^*^
		n	%	n	%	n	%	
Number of ART regimens^†^								< 0.001^‡^
	First	7	9.2	69	90.8	76	36.5	
	Second	9	19.1	38	80.9	47	22.6	
	Third	66	77.6	19	22.4	85	40.9	
Medication pick-up unit								< 0.001^‡^
	Outpatient’s	19	23.2	68	54.0	87	41.8	
	SAE/CTA^§^	63	76.8	58	46.0	121	58.2	
Reason for seeking a diagnosis								< 0.001^||^
	Opportunistic infections	3	8.6	32	91.4	35	16.8	
	Symptomatology	55	61.8	34	38.2	89	42.8	
	Rapid testing	24	28.6	60	71.4	84	40.4	
Travel time from the medication pick-up unit^¶^								< 0.001‡
	≤ 30 minutes	6	7.3	104	82.5	110	52.9	
	≥ 31 minutes	76	92.7	22	15.5	98	47.1	

Legend: *p-value = Significance level (p < 0.05);

^†^Antiretroviral therapy;

^‡^Pearson’s chi-square test;

^§^ *SAE/CTA* = Specialized Care Service/Testing and Counseling Center;

^||^Fisher’s Exact Test;

^¶^Estimated travel time via Google Maps (public transport, normal traffic), with a cutoff point of ≤ 30 min and ≥ 31 min, defined according to local characteristics and evidence of geographical barriers to access.

In the multivariate analysis using Poisson regression with robust variance (Table 4), the variables maintained as significantly associated with ART discontinuation were: male sex (PR = 1.58; 95% CI: 1.42–1.71), alcohol use disorder (PR = 1.46; 95% CI: 1.10–1.97), use of other drugs (PR = 1.98; 95% CI: 1.36–2.54), homelessness (PR = 1.38; 95% CI: 1.12–1.78), commuting time >31 minutes (PR = 1.67; 95% CI: 1.13–1.89), history of previous interruptions (PR = 1.52; 95% CI: 1.27– 1.91), and late diagnosis (PR = 1.30; 95% CI: 1.09–1.67). These results indicate that factors of social vulnerability and difficulties in access are strongly associated with treatment discontinuation.

**Table 4:** Poisson regression model with robust variance for factors associated with ART interruption among hospitalized people living with HIV (n = 208) – Santa Maria, RS, Brazil, 2022–2024.

Variables	Gross PR^*^	95% CI^†^	p-value^‡^	Adjusted PR	CI 95%	p value
Sex						
Male	1.68	1.23–2.47	0.001	1.58	1.42–1.71	< 0.033
Female	1.00			1.00		
Skin color^*^						
Black/brown	2.04	1.48–4.63	0.001	1.74	0.41–1.18	0.207
White	1.00			1.00		
Educational level						
0–8	3.81	2.17–3.97	0.001	1.10	0.95–1.28	0.547
≥ 9	1.00			1.00		
Family income						
Individual	2.14	1.85–3.74	0.001	1.85	0.58–6.32	0.085
Family	1.00			1.00		
Alcohol use disorder						
Yes	1.58	1.54–2.01	0.001	1.46	1.10–1.97	< 0.017
No	1.00			1.00		
Drug use						
Yes	2.45	1.45–2.89	0.001	1.98	1.36–2.54	< 0.001
No	1.00			1.00		
Homelessness						
Sim	1.68	1.25–1.98	0.001	1.38	1.12–1.78	< 0.005
No	1.00			1.00		
Travel time						
≤ 30 minutes	1.00			1.00		
≥ 31 minutes	2.35	1.51–3.45	0.001	1.67	1.13–1.89	< 0.001
Previous interruption history						
Yes	1.89	1.89–2.36	0.001	1.52	1.27–1.91	< 0.001
No	1.00			1.00		
Withdrawal unit						
Outpatient’s	1.48	0.95–1.42	0.001	1.16	0.58–1.21	0.358
SAE/CTA^§^	1.00			1.00		
Reason for seeking a diagnosis						
Symptoms and signs	1.85	1.12–2.11	0.001	1.30	1.09–1.67	< 0.048
Opportunistic infections	3.2	1.52–3.14	0.001	0.91	0.37–1.32	0.458
Rapid testing	1.00			1.00		

Legend: *PR = Prevalence Ratio;

^†^CI = Confidence Interval;

^‡^p-value = Significance level;

^§^SAE/CTA = Specialized Care Service/Testing and Counseling Center.

## DISCUSSION

This study identified a prevalence of 39.4% (n = 82) of ART interruption among people living with HIV who were hospitalized. Similar results were observed in a cohort study conducted in China (47.1%)^(10)^ and in research conducted in Ribeirão Preto/SP (39.3%)^(11)^. This demonstrates consistency in the findings, even across different geographical and institutional contexts. These results reinforce that, even with therapeutic advances and universal access policies, ART discontinuation remains a significant challenge in the hospital setting.

Furthermore, a Spanish study showed that interrupting ART increases the risk of hospitalization (2.92 times) and death (2.15 times), reinforcing the relevance of the problem^(12)^. These results may be related to factors such as clinical severity, presence of comorbidities, use of psychoactive substances, and disruption of the healthcare network, frequently cited in the literature as determinants of treatment interruption during hospitalization^(11,13)^.

The average interruption time identified (14.22 months; SD ± 8.04) also reflects the severity of this phenomenon. A Brazilian study demonstrates that prolonged periods of discontinuation are associated with virological failure, increased viral load, and reduced chances of achieving undetectability^(14)^, exacerbating the risk of hospitalizations and the economic impact on healthcare systems^(15)^.

Consistent with the literature, male sex has been shown to be an independent predictor of ART discontinuation (PR = 1.58; 95% CI: 1.42–1.71), a result corroborated by national and international studies^(16,17)^. Lower adherence among men may reflect patterns of masculinity that hinder self-care and engagement with health services.

Although other variables, such as skin color, low education level, and low income, showed an association in the bivariate analysis, they did not maintain significance in the adjusted model, suggesting that their effects may not be directly related to the interruption of ART.

Homelessness (PR = 1.38; 95% CI: 1.12–1.78) emerged as a major factor impacting treatment interruption. Homelessness constitutes a substantial structural barrier to the continuation of ART, and is associated with a higher risk of hospitalization and morbidity and mortality in this population^(18)^. The literature indicates that the lack of a safe place to store medications, irregular access to services, and the prioritization of basic needs represent significant challenges to adherence^(19)^. These findings reinforce the need for intersectoral policies that integrate social assistance, health, and housing.

The study confirmed that disorders related to alcohol consumption (PR = 1.46; 95% CI: 1.10–1.97) and the use of other psychoactive substances (PR = 1.98; 95% CI: 1.36–2.54) are independently associated with discontinuation of ART. Similar findings in other studies^(20,21)^ reinforce the need for integrated mental health and harm reduction actions in the care of people living with HIV. Substance use directly interferes with medication routines and therapeutic adherence, and is also associated with stigma and social marginalization, factors that require supportive and non-punitive approaches from healthcare teams.

Travel time exceeding 31 minutes to the medication pickup location (PR = 1.67; 95% CI: 1.13–1.89) was associated with ART discontinuation. This finding reinforces structural barriers, such as geographical distance and the low capillarity of dispensing services^(22)^. It is essential to optimize distribution logistics and expand the territorial coverage of *SAE/CTA*, ensuring equitable access to ART.

Furthermore, a prior history of ART interruption (PR = 1.52; 95% CI: 1.27–1.91) was a strong predictor of rediscontinuation. The recurrence of treatment abandonment highlights the need for personalized follow-up strategies, early identification, and post-hospital discharge support^(6)^.

This study identified an association between discontinuation of ART and clinical markers of severity, such as high viral load, CD4+ T lymphocyte count below 200 cells/mm^3^, emergency hospital admission, and death. These findings corroborate existing literature, which associates virological failure and advanced immunosuppression with worse prognoses^(3,5,8,23)^.

Regarding TB/HIV coinfection, although it was found to be prevalent, it did not show a statistically significant association (p = 0.092) with the interruption of ART. This result suggests that, although coinfection worsens the clinical picture, other factors, such as prior adherence and severity, may mediate this relationship. Nevertheless, the high prevalence of coinfection observed reinforces tuberculosis as a significant health problem in the context of HIV, demanding intensified screening strategies and integrated care^(24,25,26)^.

This discrepancy suggests, based on accumulated experience in clinical practice and in conducting this research, that the high number of positive sputum smear microscopy results in the group who discontinued treatment may reflect the severity of cases not being monitored by the reference service. In these scenarios, interruption of ART may have already resulted in advanced immunosuppression, increasing vulnerability to active opportunistic infections, such as tuberculosis^(25)^. This is a warning sign of failures in tracking, linking, and ensuring these individuals remain in healthcare services, which contributes to unfavorable clinical outcomes and the need for hospitalizations^(26)^. The high prevalence of coinfection in this study reinforces tuberculosis as a serious problem in Brazil, requiring urgent investments in screening, prevention, and integrated care strategies, especially among people living with HIV.

Late seeking of HIV diagnosis (PR = 1.30; 95% CI: 1.09–1.67) was also associated with interruption of ART, reflecting lower initial engagement with services and greater clinical complexity^(27)^. Initiating ART early remains one of the most effective measures to reduce morbidity, mortality, and transmission.

Hospitalization should be recognized as a strategic opportunity to restore the bond and re-engage in therapy. Strengthening the connection between hospitals, PHC, and *SAE/CTA* is essential to ensure continuity of care. Strategies such as proactive outreach, remote monitoring, multidisciplinary follow-up, and individualized discharge plans have shown potential in improving adherence^(7)^. Nursing, due to its continuous and close relationship with the patient, plays a central role in building bonds, identifying risks, and implementing personalized care plans. Comprehensive care should include restoring the dignity, identity, and autonomy of people living with HIV, promoting self-care and adherence.

One limitation of this study is the use of secondary records, which can lead to data incompleteness. The results cannot be generalized to the entire Brazilian population, as the study was conducted in a reference hospital in Rio Grande do Sul, which likely has a concentration of more severe cases.

Among its contributions, the study innovates by analyzing factors that interrupt ART in a hospitalized population, a topic that is still little explored, providing theoretical and operational support for planning nursing care and managing therapeutic adherence. Future research should delve deeper into the barriers to adherence from the users’ perspective and evaluate the impact of cross-sectoral interventions targeting vulnerable groups.

Furthermore, the findings of this study align with SDG 3 and the UNAIDS 95-95-95 target, by highlighting gaps in achieving full adherence to treatment among people living with HIV who are hospitalized. Strengthening continuous, interprofessional, and user-centered care strategies is fundamental to achieving sustained viral suppression and reducing HIV-related mortality.

## CONCLUSION

Discontinuation of antiretroviral therapy in hospitalized people living with HIV was associated with factors such as male sex, alcohol and other psychoactive substance use, homelessness, previous history of interruptions, late HIV diagnosis, and longer travel time to the medication dispensing unit. These findings reinforce the multifactorial nature of treatment adherence and the need for integrated interventions that consider social, behavioral, and clinical dimensions.

The importance of strengthening follow-up during hospitalization and ensuring continuity of care after discharge, through coordination between the different levels of the healthcare network, is highlighted. Although TB/HIV coinfection has been shown to be prevalent, it did not show a statistically significant association with ART interruption, indicating the need for further studies on its influence in this context.

The results contribute to advancing the UNAIDS 95-95-95 targets and achieving SDG 3 (Health and Well-being) by emphasizing critical points that demand innovative public policies and strategies aimed at increasing adherence, reducing interruptions, and improving the clinical and social outcomes of people living with HIV. Future research should deepen the understanding of the determinants of adherence and evaluate interventions that promote continuous and equitable care, strengthening the global commitment to ending the epidemic.

## Data Availability

The entire dataset supporting the results of this study is available upon request to the corresponding author.
