# Supplementary material for: Factors associated with antiretroviral therapy interruption in hospitalized people living with HIV: a retrospective cohort
Source: Rev Esc Enferm USP. 2026 May 22;60:e20250359. doi: 10.1590/1980-220X-REEUSP-2025-0359en (PMC13200810; doi:10.1590/1980-220X-REEUSP-2025-0359en)
Supplement: Translation

# Translation: Fatores associados à interrupção da terapia antirretroviral em pessoas vivendo com HIV hospitalizadas: coorte retrospectiva*

**DOI:** 10.1590/1980-220X-REEUSP-2025-0359pt

**Authors:** Jarbas da Silva Ziani, Laís Mara Caetano da Silva Corcini, Jenifer Härter, Caren Fabiana Alves, Stela Maris de Mello Padoin, Cláudia Zamberlan, Gabriela Pozzobon Zamberlan da Silva, Francielle Liz Monteiro

**Affiliations:** 1 Universidade Federal de Santa Maria, Centro de Ciências da Saúde, Santa Maria, RS, Brasil.; 2 Universidade Federal do Pampa, Departamento de Enfermagem, Uruguaiana, RS, Brasil.; 3 Universidade Franciscana, Departamento de Enfermagem, Santa Maria, RS, Brasil.; 4 Universidade Franciscana, Departamento de Medicina, Santa Maria, RS, Brasil.; 5 Universidade Franciscana, Departamento de Biomedicina, Santa Maria, RS, Brasil.

**Keywords:** HIV, Terapia Antirretroviral de Alta Atividade, Hospitalização, Adesão à Medicação, Fatores de Risco

## Abstract

**Objetivo:**

Identificar os fatores associados à interrupção da terapia antirretroviral (TARV) em pessoas vivendo com HIV internadas em um hospital de médio porte do Rio Grande do Sul.

**Método:**

Estudo de coorte retrospectiva e documental com 208 participantes, utilizando dados de prontuários eletrônicos de 2022 a 2024. O desfecho foi a interrupção da TARV, analisada por características sociodemográficas, clínicas e histórico terapêutico. Aplicaram-se testes Qui-quadrado, Exato de Fisher e regressão de Poisson com variância robusta (p < 0,05).

**Resultados:**

A prevalência de interrupção foi de 39,4% (IC95%: 33,0–45,8). O maior risco de interrupção foi observado entre homens (RP = 1,58; IC95%: 1,22–2,04), usuários de álcool (RP = 1,46; IC95%: 1,10–1,94), usuários de outras drogas (RP = 1,98; IC95%: 1,45–2,72), pessoas em situação de rua (RP = 1,38; IC95%: 1,08–1,76), com tempo de deslocamento superior a 31 minutos até a unidade (RP = 1,67; IC95%: 1,29–2,16) e com histórico prévio de interrupção (RP = 1,52; IC95%: 1,16–1,99).

**Conclusão:**

A interrupção da TARV relaciona-se a vulnerabilidades sociais e comportamentais, demandando ações intersetoriais e práticas de enfermagem voltadas à continuidade do cuidado e ao fortalecimento das políticas públicas de enfrentamento ao HIV.

## INTRODUÇÃO

A infecção pelo Vírus da Imunodeficiência Humana (HIV) e a síndrome da imunodeficiência adquirida (aids) permanecem como importantes problemas de saúde pública mundial. Até 2023, mais de 42,3 milhões de pessoas haviam morrido em decorrência da aids, sendo cerca de 630 mil apenas nesse ano, em grande parte por infecções oportunistas associadas à não adesão à terapia antirretroviral (TARV)^(1)^.

Com o intuito de enfrentar esse cenário, o Programa Conjunto das Nações Unidas sobre HIV/aids (UNAIDS) propôs a meta “95-95-95”, alinhada ao Objetivo de Desenvolvimento Sustentável (ODS) nº 3, que busca eliminar a epidemia até 2030^(1)^. Apesar dos avanços alcançados, o Brasil ainda não atingiu integralmente essas metas, tendo conquistado apenas o terceiro marco, relacionado à supressão viral^(2)^.

Entre 2007 e 2023, o país registrou 489.594 casos de HIV, dos quais 19,1% ocorreram na região Sul e 42.456 no Rio Grande do Sul^(2)^. Este estado mantém historicamente elevadas taxas de incidência e mortalidade, o que evidencia a necessidade de fortalecer a rede de atenção à saúde, especialmente na articulação entre a Atenção Primária à Saúde (APS) e os Serviços de Atenção Especializada (SAE), fundamentais para o acompanhamento longitudinal e o suporte terapêutico contínuo.

A TARV constitui o principal recurso terapêutico para controle da infecção pelo HIV, reduzindo a morbimortalidade, prevenindo hospitalizações e interrompendo a cadeia de transmissão por meio do conceito “Indetectável = Intransmissível (I = I)” ^(3)^. Contudo, a interrupção ou adesão irregular ao tratamento ainda representa um obstáculo significativo, influenciada por vulnerabilidades sociais, barreiras de acesso, estigma e uso de substâncias psicoativas^(4,5,6)^.

A maioria dos estudos sobre adesão à TARV concentra-se em pessoas acompanhadas em nível ambulatorial, sendo escassas as investigações que abordam a realidade de pacientes hospitalizados, grupo que apresenta maior gravidade clínica e enfrenta desafios adicionais na continuidade do cuidado^(4,7,8)^.

Dessa forma, este estudo tem como objetivo identificar os fatores associados à interrupção da terapia antirretroviral em pessoas vivendo com HIV internadas em um hospital de médio porte do Rio Grande do Sul, contribuindo para o aprimoramento das práticas assistenciais e das políticas públicas voltadas à manutenção do tratamento e à integração entre os níveis de atenção.

## MÉTODO

### Desenho do Estudo, Período e Local

Pesquisa quantitativa, do tipo coorte retrospectiva, que analisou prontuários eletrônicos de pessoas hospitalizadas com HIV admitidas no período de 1º de janeiro de 2022 a 31 de janeiro de 2024 e acompanhadas desde a internação até a alta hospitalar ou óbito, investigando a interrupção da TARV como desfecho. A descrição metodológica desta pesquisa foi orientada pelas diretrizes do *Strengthening the Reporting of Observational Studies in Epidemiology* (STROBE).

A pesquisa foi realizada em uma unidade de internação clínica de um hospital-escola situado no estado do RS, Brasil. A escolha do cenário deu-se em razão de a instituição ser referência no atendimento às pessoas com doenças infectocontagiosas. A instituição possui caráter público e filantrópico, sendo referência para 33 municípios da macrorregião Centro-Oeste do RS. Essa macrorregião abrange uma população estimada em mais de 500 mil habitantes. Com relação à unidade de desenvolvimento do estudo, ela é composta por 34 leitos, sendo 15 masculinos, 15 femininos e quatro de isolamento.

### População, Critérios de Inclusão e Exclusão

A população foi composta por pessoas com diagnóstico de HIV admitidas na unidade de internação no período de 1º de janeiro de 2022 a 31 de janeiro de 2024. Esse período foi estabelecido porque, durante os anos de 2020 a 2021, a unidade tornou-se referência para o atendimento de pessoas com COVID-19. Dessa forma, buscou-se minimizar os efeitos da pandemia nos dados obtidos e analisados.

Os critérios de inclusão foram: prontuários eletrônicos de pessoas com mais de 18 anos, diagnosticadas com HIV e internadas por no mínimo 72 horas. Esse tempo foi considerado com o objetivo de obter as informações mínimas necessárias ao estudo. O seguimento foi realizado desde a admissão hospitalar até a alta ou o óbito. Os critérios de exclusão foram: prontuários não localizados ou que não apresentavam informações suficientes; prontuários nos quais o diagnóstico de HIV foi descartado ou permaneceu inconclusivo durante a hospitalização; e pessoas que iniciaram a TARV durante a internação, uma vez que o estudo foca na interrupção do tratamento antes desse evento. Não foram coletados dados de pessoas reinternadas após o período do estudo.

Considerou-se uma amostra mínima de 188 prontuários, calculada com base no total de internações por HIV na unidade nos últimos cinco anos, adotando-se tolerância de 5% ao erro, grau de confiança de 95% e proporção da característica de interesse de 0,5 (50%), valor conservador escolhido por não se conhecer a prevalência exata das interrupções de terapia nessa população hospitalar.

### Coleta de Dados

A coleta de dados ocorreu entre agosto de 2023 e abril de 2024, sendo conduzida pelo autor da pesquisa e por uma coletadora voluntária, ambos previamente treinados para minimizar quaisquer vieses de coleta.

Para a coleta dos dados foi realizada uma busca na base eletrônica de prontuários da instituição, abrangendo todas as pessoas admitidas na unidade de internação durante o período do estudo. Foram identificadas 3.325 internações e, após a exclusão de duplicatas, foram incluídos 3.258 prontuários. Em seguida, procedeu-se à identificação dos prontuários de pessoas que vivem com HIV. Para essa etapa, analisaram-se na íntegra os prontuários para selecionar os que atendiam aos critérios de inclusão do estudo, totalizando 248 prontuários de pessoas que vivem com HIV. Após a aplicação dos critérios de exclusão, obteve-se a amostra final de 208 participantes, sendo que 40 prontuários foram excluídos do estudo (15 por pertencerem a internações com tempo inferior a 72 horas, 14 por não apresentarem informações suficientes para as variáveis estudadas e 11 por terem iniciado o uso da TARV durante a hospitalização). O fluxo amostral detalhado também é apresentado visualmente na Figura 1.

**Figura 1:** Fluxograma do número de pessoas elegíveis para o estudo (n = 208) – Santa Maria, RS, Brasil, 2022–2024.

Fonte: Elaborado pelo autor (2025).

### Instrumento de Coleta de Dados e Variáveis

O instrumento foi elaborado com base nas recomendações do Protocolo Clínico e Diretrizes Terapêuticas para Manejo da Infecção pelo HIV em adultos do Ministério da Saúde (MS) do ano de 2018 e foi composto pelas variáveis sociodemográficas sexo (masculino/feminino), cor da pele (branca e preta/parda), escolaridade (menos de oito anos de estudo e mais de oito anos de estudo), classe econômica. A estratificação da classe econômica foi realizada de acordo com a classificação já utilizada pela instituição onde o estudo foi realizado, que categoriza a renda mensal familiar dos participantes da seguinte forma: Classe A: renda mensal superior a R$ 11.262, Classe B: entre R$ 8.641 e R$ 11.261; Classe C: entre R$ 2.005 e R$ 8.640; Classe D: entre R$ 1.255 e R$ 2.004; Classe E: entre R$ 0,00 e R$ 1.254. Incluíram-se também orientação sexual (homossexual, heterossexual e bissexual), estado civil (solteiro e casado) e parceria fixa (sim ou não). Quanto aos fatores de vulnerabilidade social e comportamental, foram incluídas as seguintes variáveis dicotômicas (sim ou não): transtorno relacionado ao consumo de tabaco, transtorno relacionado ao consumo de álcool, pessoas em situação de rua, uso de drogas e/ou álcool, e privação de liberdade.

A variável clínica foi aids (sim ou não). Para a classificação dessa variável, adotaram-se os critérios do MS. O caso foi considerado como aids quando havia registro explícito do diagnóstico no prontuário ou quando atendia a critérios laboratoriais (LT-CD4+ <200 células/mm^3^ ou carga viral >1.000 cópias/mL^(3)^) e/ou clínicos compatíveis (piora do quadro associada a infecções oportunistas). Na ausência de dados laboratoriais, prevaleceu o critério clínico e/ou o registro do CID, de modo a reduzir a perda de casos.

Considerou-se, ainda, a forma de admissão hospitalar (eletiva ou urgente), coinfecção (tuberculose/HIV), carga viral (indetectável, baixa e alta). A carga viral foi categorizada em: indetectável, até 200 cópias/mL (sem risco de transmissão sexual); baixa, entre 200 e 1.000 cópias/mL (risco quase nulo de transmissão sexual); alta, superior a 1.000 cópias/mL (risco relevante de transmissão sexual)^(2)^; e os LT-CD4+ (LT-CD4+ maior de 201 células/mm^3^ e LT-CD4+ menor de 200 células/mm^3^). Além disso, foram considerados o histórico de internações anteriores (sim ou não) e o desfecho da internação (alta hospitalar ou óbito). Ressalta-se que o instrumento foi compartilhado com um comitê de cinco especialistas na temática do HIV e epidemiologia, sendo avaliado e validado quanto ao seu conteúdo e semântica por meio do cálculo do Índice de Validade de Conteúdo (IVC), com o intuito de aprimorá-lo. Itens com IVC inferior a 0,80 foram revisados ou removidos.

A amostra foi estratificada em dois grupos, considerando a variável interrupção do tratamento, que foi analisada com base no critério de tempo. O MS define a interrupção como a ausência de retirada da medicação por período superior a 28 dias após a data prevista para a última dispensação, associada ao afastamento do acompanhamento ambulatorial^(3)^. Ressalta-se que essa variável estava disponível no prontuário do paciente, pois o SAE do município compartilha essa informação com o hospital.

Em relação ao histórico terapêutico, foram consideradas as seguintes variáveis: histórico de interrupção anterior da TARV (sim ou não), número de esquemas da TARV (primeiro, segundo e terceiro ou mais esquemas), unidade de retirada da medicação (ambulatorial ou Serviço de Atendimento Especializado/Centro de Testagem e Aconselhamento – SAE/CTA), motivo da busca pelo diagnóstico (infecções oportunistas, sintomatologia ou testagem rápida) e tempo de distância da unidade de retirada da medicação (inferior a 30 minutos e superior a 31 minutos).

Para estruturar a variável tempo de deslocamento até a unidade de retirada da medicação, utilizou-se o endereço de residência e a unidade de referência da pessoa. Para estimar o tempo, a variável foi mensurada na ferramenta *Google Maps*, levando em consideração o trajeto por meio de transporte coletivo. Especificamente, foi utilizado o tempo médio estimado de deslocamento em condições normais de tráfego, evitando a flutuação do trânsito real, que é difícil de padronizar retrospectivamente.

A estratificação do tempo de deslocamento (≤30 minutos e ≥31 minutos) foi definida considerando as características geográficas e de mobilidade do município, nas quais deslocamentos acima de 30 minutos representam uma barreira de acesso frequentemente observada nos trajetos urbanos. Assim, o ponto de corte reflete tanto evidências da literatura sobre barreiras geográficas ao acesso aos serviços de saúde quanto a realidade local de deslocamento urbano^(4,7)^.

### Análise dos Dados

Elaborou-se um banco de dados em planilha do *Excel® (Microsoft, versão 10, EUA)*, com dupla digitação, seguida por uma validação para minimizar o viés de aferição e garantir a fidedignidade dos dados. Após a validação da planilha e realização de correções, os dados foram transferidos para um banco definitivo, o qual foi importado para análise no *software Statistical Package for the Social Sciences* (SPSS), versão 20.0.

Para a análise dos dados, utilizou-se estatística descritiva para caracterização da amostra. Para as variáveis categóricas, foram apresentadas frequência absoluta (n) e relativa (%). Para as variáveis numéricas, a normalidade da distribuição foi verificada por meio do teste de *Kolmogorov-Smirnov*. Variáveis com distribuição normal foram descritas pela média e desvio padrão (DP±), enquanto variáveis com distribuição assimétrica foram apresentadas pela mediana e pelo intervalo interquartil.

A associação entre as variáveis categóricas foi avaliada por meio dos testes Qui-Quadrado de Pearson e Teste Exato de Fisher, quando apropriado, ou seja, quando as premissas do Qui-Quadrado não eram atendidas, como células com contagens esperadas menores que cinco, por exemplo. Foi considerado um nível de significância estatística de 5% (p < 0,05).

Também foi realizada uma análise multivariada conduzida por meio da regressão de Poisson com variância robusta, com o objetivo de identificar os fatores associados à interrupção da terapia antirretroviral. Antes da construção dos modelos, a multicolinearidade entre as variáveis preditoras foi verificada utilizando o Fator de Inflação da Variância (VIF), com valores aceitáveis abaixo de 5. As variáveis foram inseridas em blocos temáticos sucessivos: sociodemográfico (Bloco 1), vulnerabilidades sociais e comportamentais (Bloco 2) e histórico clínico/terapêutico (Bloco 3). As variáveis com valor de p < 0,20 na análise bivariada foram consideradas para inclusão no modelo inicial de cada bloco, critério recomendado pela literatura científica para maximizar a chance de inclusão de variáveis relevantes no modelo multivariado^(9)^. A permanência no modelo final foi definida com base na significância estatística (p < 0,05) e relevância epidemiológica.

O modelo conceitual deste estudo postula que a interrupção da TARV é influenciada por vulnerabilidades sociais, comportamentais, e histórico clínico/terapêutico. O desfecho principal, interrupção da TARV, foi definido conforme os critérios do MS. Para a construção do modelo causal, foram consideradas potenciais variáveis confundidoras, amplamente evidenciadas na literatura como influenciadoras do desfecho de interrupção da TARV, incluindo carga viral, LT CD4+, diagnóstico de aids, desfecho da internação, forma de admissão hospitalar e interrupção prévia e coinfecção tuberculose-HIV^(1,3,4,7,8)^. O impacto dessas variáveis confundidoras foi ajustado nas análises multivariadas para melhor estimar a associação entre os fatores de interesse e a interrupção da TARV.

Este estudo possui algumas limitações inerentes ao seu desenho retrospectivo. Primeiro, a dependência de registros em prontuários pode levar a um viés de informação (subnotificação), já que a qualidade e completude dos dados dependem do registro clínico. Segundo, o estudo foi realizado em um hospital de referência que concentra casos mais graves, o que pode gerar um viés de seleção e limitar a generalização dos resultados para outras populações de pessoas com HIV. Por fim, o uso de dados secundários impede o estabelecimento definitivo de causalidade.

### Aspectos Éticos

O estudo foi conduzido conforme as diretrizes éticas nacionais e internacionais, sendo aprovado pelo Comitê de Ética em Pesquisa da Universidade Franciscana sob o parecer nº 5.828.312. Quanto ao Termo de Consentimento Livre e Esclarecido, este foi dispensado, visto que os dados foram obtidos por meio de prontuários eletrônicos.

## RESULTADOS

Foram elegíveis 248 prontuários de pessoas vivendo com HIV hospitalizadas no período do estudo. Destes, 40 foram excluídos (15 por internações inferiores a 72 horas, 14 por informações insuficientes e 11 por início de TARV durante a hospitalização). Assim, a amostra final foi composta por 208 prontuários, sendo 82 (39,4%) com interrupção da TARV e 126 (60,6%) sem interrupção.

A média de idade dos participantes foi de 39,6 anos (DP ± 13,2; variação 18–89), sendo que 82 (39,4%) apresentaram interrupção da TARV. O tempo médio de interrupção foi de 14,2 meses (DP ± 8,0; mediana 12; intervalo 5–47 meses).

Na análise bivariada, observou-se associação significativa entre a interrupção da TARV e sexo masculino, cor preta/parda, menor escolaridade (<8 anos), classe econômica E, renda individual e ausência de parceria fixa (p < 0,001), conforme Tabela 1. As demais variáveis sociodemográficas não apresentaram associação estatisticamente significativa.

**Tabela 1:** Características sociodemográficas das pessoas que vivem com HIV hospitalizadas, segundo interrupção da TARV (n = 208) – Santa Maria, RS, Brasil, 2022–2024.

Variáveis	Categoria	Interrupção		Não interrupção		Total		p-valor^* †^
		n	%	n	%	n	%	
Sexo								< 0,001^†^
	Masculino	66	80,5	58	46,0	124	59,6
	Feminino	16	19,5	68	54,0	84	40,4	
Cor autodeclarada								< 0,001^†^
	Preta/Parda	67	81,7	52	41,3	119	57,2	
	Branca	15	18,3	74	58,7	89	42,8	
Nível de escolaridade								< 0,001^†^
	0–8	66	80,5	27	21,4	93	44,7	
	≥ 9	16	19,5	99	78,6	115	55,3	
Classe econômica^§^								< 0,001^‡^
	Classe C	1	1,9	52	98,1	53	25,5	
	Classe D	14	22,6	48	77,4	62	29,8	
	Classe E	67	72,0	26	28,0	93	44,7	
Renda mensal								< 0,001^†^
	Individual	72	87,8	41	32,5	113	54,3	
	Familiar	10	12,2	85	67,5	95	45,7	
Orientação sexual								0,441^†^
	Homossexual	27	45,8	32	54,2	59	28,4	
	Heterossexual	46	36,6	87	65,4	113	63,9	
	Bissexual	9	56,2	7	43,8	16	7,7	
Estado civil								< 0,001^†^
	Solteiro(a)	74	90,2	36	28,6	110	52,9	
	Casado(a)	8	9,8	90	71,4	98	47,1	
Parceria Fixa								< 0,001^‡^
	Sim	4	4,9	90	71,4	94	45,2
	Não	78	95,1	36	28,6	114	54,8	

Legenda: *p-valor = Nível de significância (p < 0,05);

^†^ = Teste qui-quadrado de Pearson;

^‡^ = Teste de Exato de Fisher;

^§^ = Estratificação da classe econômica: realizada conforme classificação utilizada pela instituição: Classe A = renda mensal > R$ 11.262; Classe B = R$ 8.641–11.261; Classe C = R$ 2.005–8.640; Classe D = R$ 1.255– 2.004; Classe E = R$ 0–1.254. Nenhum participante foi classificado nas classes A ou B.

A interrupção da TARV apresentou associação estatisticamente significativa com fatores de vulnerabilidade social, comportamental e clínica. Entre as variáveis comportamentais, observou-se maior prevalência de interrupção entre pessoas que faziam uso de tabaco (86,6%), consumiam álcool (87,8%), utilizavam outras drogas (74,4%) e viviam em situação de rua (42,7%) (p < 0,001). Não foi identificada associação significativa com a variável privação de liberdade (p = 0,378).

Entre os fatores clínicos, a interrupção da TARV esteve associada à presença de diagnóstico de aids (86,6%), carga viral elevada (95,1%) e contagem de LT-CD4+ inferior a 200 células/mm^3^ (89,0%) (p < 0,001), internações de caráter urgente (81,7%). O histórico de internações anteriores também apresentou associação significativa, sendo observado em 79,3% daqueles que interromperam o tratamento (p < 0,001). Em relação ao desfecho da internação, a interrupção foi mais prevalente entre os pacientes que evoluíram para óbito (65,9%), em comparação com os que receberam alta hospitalar, diferença estatisticamente significativa (p < 0,001). A coinfecção tuberculose/HIV não apresentou associação significativa com a interrupção da TARV (p = 0,092), conforme exposto na Tabela 2.

**Tabela 2:** Fatores de vulnerabilidade e condições clínicas associadas à interrupção da TARV em pessoas vivendo com HIV hospitalizadas (n = 208) – Santa Maria, RS, Brasil, 2022–2024.

Variáveis	Categoria	Interrupção		Não interrupção		Total		p-valor^*^
		n	%	n	%	n	%	
Bloco 1 – Fatores de vulnerabilidade								< 0,001^†^
Consumo de tabaco	Consome	71	86,6	40	31,7	111	53,4	
	Não consome	11	13,4	86	68,3	97	46,6	
Consumo de álcool								< 0,001^†^
	Consome	72	87,8	30	23,8	102	49,0	
	Não consome	10	12,2	96	76,2	106	51,0	
Consumo de outras drogas								< 0,001^†^
	Consome	61	74,4	13	10,3	74	35,6	
	Não consome	21	25,6	113	89,7	134	64,4	
Situação de rua								< 0,001^†^
	Sim	35	42,7	10	7,9	45	21,6	
	Não	47	57,3	116	92,1	163	78,4	
Privado de liberdade								
	Sim	5	6,1	12	9,5	17	8,2	0,378^‡^
	Não	77	93,9	114	90,5	191	91,8	
Bloco 2 – Condições clínicas								
Diagnóstico de aids^§^								< 0,001^†^
	Sim	71	86,6	27	21,4	98	47,1	
	Não	11	13,4	99	78,6	110	52,9	
Coinfecção HIV/Tuberculose								0,092^†^
	Sim	61	74,4	20	15,9	81	38,9	
	Não							
Bloco 3 – Exames laboratoriais								
Carga Viral^||^								< 0,001^‡^
	Alta	78	95,1	53	42,1	131	63,0	
	Baixa	3	3,7	37	29,4	40	19,2	
	Indetectável	1	1,2	36	28,6	37	17,8	
LT CD4+^¶^								< 0,001^†^
	< 200	73	89,0	52	41,3	125	60,1	
	> 201	9	11,0	74	58,7	83	39,9	
Bloco 4 – Histórico e desfecho da hospitalização								
Caráter da internação								< 0,001^†^
	Urgente	65	81,7	40	31,7	107	51,4	
	Eletiva	15	18,3	86	68,3	101	48,6	
Histórico de internações anteriores								< 0,001^†^
	Sim	65	79,3	37	29,4	102	49,0	
	Não	17	20,7	89	70,6	106	51,0	
Desfecho da hospitalização								< 0,001^†^
	Alta hospitalar	28	34,1	106	84,1	134	64,4	
	Óbito	54	65,9	20	15,9	74	35,6	

Legenda: *p-valor = Nível de significância (p < 0,05);

^†^ = Teste qui-quadrado de Pearson;

^‡^ = Teste de Exato de Fisher;

^§^ = O diagnóstico de aids foi definido conforme critérios do MS: presença de infecções oportunistas, carga viral >1.000 cópias/mL, LT-CD4+ <200 células/mm^3^ ou registro com CID correspondente;

^||^ = A carga viral foi categorizada como: indetectável (≤200 cópias/mL), baixa (200–1.000 cópias/mL) e alta (>1.000 cópias/mL);

^¶^ = Linfócitos T CD4+, considerou-se baixo quando ≤ 200 células/mm^3^ e alto quando > 200 células/mm^3^.

Quanto ao histórico terapêutico, a interrupção da TARV demonstrou associação estatística com o número de esquemas de TARV (com maior frequência de interrupção no terceiro ou maior quantidade de esquemas realizados), e a unidade de retirada da medicação (SAE/CTA) (Tabela 3). Adicionalmente, a interrupção foi mais prevalente entre pessoas que buscaram o diagnóstico de HIV devido a infecções oportunistas ou sintomatologia, e aqueles que demoravam mais de 31 minutos para se deslocar até a unidade de retirada da medicação.

**Tabela 3:** Histórico terapêutico entre pessoas vivendo com HIV hospitalizadas, segundo interrupção da TARV (n = 208) – Santa Maria, RS, Brasil, 2022–2024

Variáveis	Categoria	Interrupção		Não interrupção		Total		p-valor^*^
		n	%	n	%	n	%	
Número de esquemas da TARV^†^								< 0,001^‡^
	Primeiro	7	9,2	69	90,8	76	36,5	
	Segundo	9	19,1	38	80,9	47	22,6	
	Terceiro	66	77,6	19	22,4	85	40,9	
Unidade de retirada da medicação								< 0,001^‡^
	Ambulatorial	19	23,2	68	54,0	87	41,8	
	SAE/CTA^§^	63	76,8	58	46,0	121	58,2	
Motivo da busca pelo diagnóstico								< 0,001^||^
	Infecções oportunistas	3	8,6	32	91,4	35	16,8	
	Sintomatologia	55	61,8	34	38,2	89	42,8	
	Testagem rápida	24	28,6	60	71,4	84	40,4	
Tempo de distância da unidade de retirada da medicação^¶^								< 0,001^‡^
	≤ 30 minutos	6	7,3	104	82,5	110	52,9
	≥ 31 minutos	76	92,7	22	15,5	98	47,1	

Legenda: *p-valor = Nível de significância (p < 0,05);

^†^ = Terapia Antirretroviral;

^‡^ = Teste qui-quadrado de Pearson;

^§^SAE/CTA = Serviço Atenção Especializada/Centro de Testagem e Aconselhamento;

^||^ = Teste de Exato de Fisher;

^¶^ = Tempo de deslocamento estimado via Google Maps (transporte coletivo, tráfego normal), com ponto de corte ≤30 min e ≥31 min, definido conforme características locais e evidências sobre barreiras geográficas ao acesso.

Na análise multivariada por Regressão de Poisson com variância robusta (Tabela 4), as variáveis mantidas como significativamente associadas à interrupção da TARV foram: sexo masculino (RP = 1,58; IC95%: 1,42–1,71), transtorno relacionado ao uso de álcool (RP = 1,46; IC95%: 1,10–1,97), uso de outras drogas (RP = 1,98; IC95%: 1,36–2,54), situação de rua (RP = 1,38; IC95%: 1,12–1,78), tempo de deslocamento >31 minutos (RP = 1,67; IC95%: 1,13–1,89), histórico de interrupções anteriores (RP = 1,52; IC95%: 1,27–1,91) e diagnóstico tardio (RP = 1,30; IC95%: 1,09–1,67). Esses resultados indicam que fatores de vulnerabilidade social e dificuldades de acesso estão fortemente associados à descontinuidade do tratamento.

**Tabela 4:** Modelo de Regressão de Poisson com variância robusta para os fatores associados à interrupção da TARV entre pessoas vivendo com HIV hospitalizadas (n = 208) – Santa Maria, RS, Brasil, 2022–2024.

Variáveis	RP^*^ bruta	IC^†^ 95%	p-valor^‡^	RP ajustado	IC 95%	p-valor
Sexo						
Masculino	1,68	1,23–2,47	0,001	1,58	1,42–1,71	< 0,033
Feminino	1,00			1,00		
Cor da pele						
Preta/Parda	2,04	1,48–4,63	0,001	1,74	0,41–1,18	0,207
Branca	1,00			1,00		
Nível de escolaridade						
0–8	3,81	2,17–3,97	0,001	1,10	0,95–1,28	0,547
≥ 9	1,00			1,00		
Renda familiar						
Individual	2,14	1,85–3,74	0,001	1,85	0,58–6,32	0,085
Familiar	1,00			1,00		
Transtorno relacionado ao uso de álcool						
Sim	1,58	1,54–2,01	0,001	1,46	1,10–1,97	< 0,017
Não	1,00			1,00		
Uso de drogas						
Sim	2,45	1,45–2,89	0,001	1,98	1,36–2,54	< 0,001
Não	1,00			1,00		
Situação de rua						
Sim	1,68	1,25–1,98	0,001	1,38	1,12–1,78	< 0,005
Não	1,00			1,00		
Tempo de deslocamento						
≤ 30 minutos	1,00			1,00		
≥ 31 minutos	2,35	1,51–3,45	0,001	1,67	1,13–1,89	< 0,001
Histórico de interrupção anterior						
Sim	1,89	1,89–2,36	0,001	1,52	1,27–1,91	< 0,001
Não	1,00			1,00		
Unidade de retirada						
Ambulatório	1,48	0,95–1,42	0,001	1,16	0,58–1,21	0,358
SAE/CTA^§^	1,00			1,00		
Motivo da busca pelo diagnóstico						
Sinais e sintomas	1,85	1,12–2,11	0,001	1,30	1,09–1,67	< 0,048
Infecções oportunistas	3,2	1,52–3,14	0,001	0,91	0,37–1,32	0,458
Testagem rápida	1,00			1,00		

Legenda: *RP = Razão de Prevalência;

^†^IC = Intervalo de Confiança;

^‡^p-valor = Nível de significância;

^§^SAE/CTA = Serviço Atenção Especializada/Centro de Testagem e Aconselhamento.

## DISCUSSÃO

Este estudo identificou uma prevalência de 39,4% (n = 82) de interrupção da TARV entre pessoas vivendo com HIV hospitalizadas. Resultados semelhantes foram observados em um estudo de coorte realizado na China (47,1%)^(10)^ e em pesquisa conduzida em Ribeirão Preto/SP (39,3%)^(11)^, o que demonstra consistência nos achados, mesmo que em diferentes contextos geográficos e institucionais. Tais resultados reforçam que, mesmo diante de avanços terapêuticos e políticas de acesso universal, a interrupção da TARV permanece um desafio expressivo no contexto hospitalar.

Além disso, estudo espanhol evidenciou que a interrupção da TARV aumenta o risco de hospitalização (2,92 vezes) e óbito (2,15 vezes), reforçando a relevância do problema^(12)^.Tais resultados podem estar relacionados a fatores como gravidade clínica, presença de comorbidades, uso de substâncias psicoativas e desarticulação da rede de atenção à saúde, frequentemente apontados pela literatura como determinantes da interrupção do tratamento durante a hospitalização^(11,13)^.

O tempo médio de interrupção identificado (14,22 meses; DP ± 8,04) também reflete na gravidade desse fenômeno. Estudo brasileiro demonstra que períodos prolongados de descontinuação estão associados à falha virológica, elevação da carga viral e redução das chances de alcançar a indetectabilidade^(14)^, agravando o risco de hospitalizações e o impacto econômico sobre os sistemas de saúde^(15)^.

Em consonância com a literatura, o sexo masculino demonstrou ser um preditor independente de interrupção da TARV (RP = 1,58; IC 95%: 1,42–1,71), resultado corroborado por estudos nacionais e internacionais^(16,17)^. A menor adesão entre homens pode refletir padrões de masculinidade que dificultam o autocuidado e o vínculo com os serviços de saúde.

Embora outras variáveis, como cor da pele, baixa escolaridade e baixa renda, tenham apresentado associação na análise bivariada, essas não mantiveram significância no modelo ajustado, sugerindo que seus efeitos podem não estar diretamente relacionados a interrupção da TARV.

A situação de rua (RP = 1,38; IC 95%: 1,12–1,78) emergiu como um fator de grande impacto na interrupção do tratamento. A falta de moradia configura-se como uma barreira estrutural substancial para a continuidade da TARV, estando associada a maior risco de hospitalização e morbimortalidade nessa população^(18)^. A literatura aponta que a ausência de local seguro para armazenamento de medicamentos, o acesso irregular aos serviços e a priorização de necessidades básicas representam desafios significativos à adesão^(19)^. Esses achados reforçam a necessidade de políticas intersetoriais que integrem assistência social, saúde e habitação.

O estudo confirmou que transtornos relacionados ao consumo de álcool (RP = 1,46; IC 95%: 1,10–1,97) e ao uso de outras substâncias psicoativas (RP = 1,98; IC 95%: 1,36–2,54) são independentemente associados à interrupção da TARV. Achados semelhantes em outras pesquisas^(20,21)^ reforçam a necessidade de ações integradas de saúde mental e redução de danos no cuidado a pessoas que vivem com HIV. O uso de substâncias interfere diretamente na rotina medicamentosa e na adesão terapêutica, além de estar associado ao estigma e à marginalização social, fatores que exigem abordagens acolhedoras e não punitivas por parte das equipes de saúde.

O tempo de deslocamento superior a 31 minutos até o local de retirada dos medicamentos (RP = 1,67; IC 95%: 1,13–1,89) esteve associado à interrupção da TARV. Esse achado reforça barreiras estruturais, como a distância geográfica e a baixa capilaridade dos serviços de dispensação^(22)^. Torna-se essencial otimizar a logística de distribuição e ampliar a cobertura territorial dos SAE/CTA, garantindo o acesso equitativo à TARV.

Além disso, o histórico prévio de interrupção da TARV (RP = 1,52; IC 95%: 1,27–1,91) foi um forte preditor de nova descontinuação. A reincidência do abandono terapêutico evidencia a necessidade de estratégias personalizadas de seguimento, identificação precoce e apoio pós-alta hospitalar^(6)^.

O presente estudo identificou associação entre a interrupção da TARV e marcadores clínicos de gravidade, como carga viral elevada, contagem de linfócitos T CD4+ inferior a 200 células/mm^3^, internação hospitalar em caráter de urgência e ocorrência de óbito. Esses achados corroboram a literatura existente, que associa falha virológica e imunossupressão avançada a piores prognósticos^(3,5,8,23)^.

Quanto à coinfecção TB/HIV, embora tenha se mostrado prevalente, não apresentou associação estatisticamente significativa (p = 0,092) com a interrupção da TARV. Esse resultado sugere que, embora a coinfecção agrave o quadro clínico, outros fatores, tais como adesão prévia e gravidade podem mediar essa relação. Ainda assim, a elevada prevalência de coinfecção observada reforça a tuberculose como um importante agravo no contexto do HIV, demandando intensificação das estratégias de rastreamento e integração do cuidado^(24,25,26)^.

Essa discrepância sugere, com base na experiência acumulada na prática clínica e na condução desta pesquisa, que a elevada quantidade de baciloscopias de escarro positivas no grupo em interrupção pode refletir a gravidade dos casos desacompanhados pelo serviço de referência. Nesses cenários, a interrupção da TARV pode já ter resultado em imunossupressão avançada, aumentando a vulnerabilidade a infecções oportunistas ativas, como a tuberculose^(25)^. Trata-se de um sinal de alerta para falhas no rastreamento, vinculação e permanência dessas pessoas nos serviços de saúde, o que contribui para a evolução clínica desfavorável e necessidade de hospitalizações^(26)^. A elevada prevalência da coinfecção nesse estudo reforça a tuberculose como um grave problema no Brasil, exigindo investimentos urgentes em estratégias de rastreamento, prevenção e cuidado integrado, sobretudo entre pessoas vivendo com HIV.

A busca tardia pelo diagnóstico do HIV (RP = 1,30; IC 95%: 1,09–1,67) também se associou à interrupção da TARV, refletindo menor engajamento inicial com os serviços e maior complexidade clínica^(27)^. Iniciar precocemente a TARV continua sendo uma das medidas mais efetivas para reduzir morbimortalidade e transmissão.

A internação hospitalar deve ser reconhecida como uma oportunidade estratégica para o resgate do vínculo e a reengajamento terapêutico. O fortalecimento da articulação entre hospitais, APS e SAE/CTA é essencial para garantir continuidade do cuidado. Estratégias como busca ativa, telemonitoramento, acompanhamento multiprofissional e planos de alta individualizados têm demonstrado potencial na melhoria da adesão^(7)^. A enfermagem, por seu papel contínuo e próximo ao paciente, tem posição central na construção de vínculos, identificação de riscos e implementação de planos de cuidado personalizados. O cuidado integral deve incluir o resgate da dignidade, da identidade e da autonomia das pessoas vivendo com HIV, favorecendo o autocuidado e a adesão.

Como limitação desta pesquisa, destaca-se o uso de registros secundários, o que pode gerar incompletude de dados. Os resultados não são generalizáveis para toda a população brasileira, pois o estudo foi conduzido em um hospital de referência no Rio Grande do Sul, com provável concentração de casos mais graves.

Entre as contribuições, o estudo inova ao analisar fatores de interrupção da TARV em população hospitalizada, um tema ainda pouco explorado, fornecendo subsídios teóricos e operacionais para o planejamento do cuidado de enfermagem e gestão da adesão terapêutica. Futuras pesquisas devem aprofundar as barreiras à adesão sob a perspectiva dos usuários e avaliar o impacto de intervenções intersetoriais voltadas a grupos vulneráveis.

Para mais, os achados deste estudo dialogam com o ODS 3 e com a meta UNAIDS 95-95-95, ao evidenciar lacunas no alcance da adesão plena ao tratamento entre pessoas vivendo com HIV hospitalizadas. O fortalecimento das estratégias de cuidado contínuo, interprofissional e centrado no usuário é fundamental para alcançar a supressão viral sustentada e reduzir a mortalidade associada ao HIV.

## CONCLUSÃO

A interrupção da terapia antirretroviral em pessoas vivendo com HIV hospitalizadas esteve associada a fatores como sexo masculino, uso de álcool e outras substâncias psicoativas, situação de rua, histórico prévio de interrupções, diagnóstico tardio do HIV e maior tempo de deslocamento até a unidade de dispensação da medicação. Esses achados reforçam a natureza multifatorial da adesão ao tratamento e a necessidade de intervenções integradas que considerem dimensões sociais, comportamentais e clínicas.

Destaca-se a importância de fortalecer o acompanhamento durante a internação e garantir a continuidade do cuidado após a alta, por meio da articulação entre os diferentes níveis da rede de atenção à saúde. Embora a coinfecção TB/HIV tenha se mostrado prevalente, não apresentou associação estatisticamente significativa com a interrupção da TARV, o que indica a necessidade de estudos adicionais sobre sua influência nesse contexto.

Os resultados contribuem para o avanço das metas UNAIDS 95-95-95 e para o alcance do ODS 3 (Saúde e Bem-Estar), ao evidenciar pontos críticos que demandam políticas públicas e estratégias inovadoras voltadas à ampliação da adesão, à redução das interrupções e à melhoria dos desfechos clínicos e sociais das pessoas que vivem com HIV. Futuras pesquisas devem aprofundar a compreensão dos determinantes da adesão e avaliar intervenções que promovam cuidado contínuo e equitativo, fortalecendo o compromisso global com o fim da epidemia.

## Data Availability

Todo o conjunto de dados que dá suporte aos resultados deste estudo está disponível mediante solicitação ao autor correspondente.

# Translation: Factores asociados a la interrupción de la terapia antirretroviral en personas hospitalizadas con VIH: un estudio de cohorte retrospectivo^*^

**DOI:** 10.1590/1980-220X-REEUSP-2025-0359es

**Authors:** Jarbas da Silva Ziani, Laís Mara Caetano da Silva Corcini, Jenifer Härter, Caren Fabiana Alves, Stela Maris de Mello Padoin, Cláudia Zamberlan, Gabriela Pozzobon Zamberlan da Silva, Francielle Liz Monteiro

**Affiliations:** 1 Universidade Federal de Santa Maria, Centro de Ciências da Saúde, Santa Maria, RS, Brasil.; 2 Universidade Federal do Pampa, Departamento de Enfermagem, Uruguaiana, RS, Brasil.; 3 Universidade Franciscana, Departamento de Enfermagem, Santa Maria, RS, Brasil.; 4 Universidade Franciscana, Departamento de Medicina, Santa Maria, RS, Brasil.; 5 Universidade Franciscana, Departamento de Biomedicina, Santa Maria, RS, Brasil.

**Keywords:** VIH, Terapia Antirretroviral Altamente Activa, Hospitalización, Cumplimiento de la Medicación, Factores de Riesgo

## Abstract

**Objetivo:**

Identificar los factores asociados a la interrupción de la terapia antirretroviral (TAR) en personas que viven con el VIH hospitalizadas en un hospital de tamaño mediano en Rio Grande do Sul.

**Método:**

Estudio de cohorte retrospectivo y documental con 208 participantes, utilizando datos de historias clínicas electrónicas desde 2022 hasta 2024. El resultado fue la interrupción del tratamiento antirretroviral, analizado según características sociodemográficas, clínicas e historial terapéutico. Se aplicaron la prueba de chi-cuadrado, la prueba exacta de Fisher y la regresión de Poisson con varianza robusta (p < 0,05).

**Resultados:**

La prevalencia de interrupción fue del 39,4% (IC del 95%: 33,0–45,8). El mayor riesgo de interrupción se observó entre los hombres (PR = 1,58; IC del 95%: 1,22–2,04), los consumidores de alcohol (PR = 1,46; IC del 95%: 1,10–1,94), los consumidores de otras drogas (PR = 1,98; IC del 95%: 1,45–2,72), las personas sin hogar (PR = 1,38; IC del 95%: 1,08–1,76), aquellos con un tiempo de viaje superior a 31 minutos hasta la unidad (PR = 1,67; IC del 95%: 1,29–2,16) y aquellos con antecedentes de interrupción (PR = 1,52; IC del 95%: 1,16–1,99).

**Conclusión:**

La interrupción de la TAR está relacionada con vulnerabilidades sociales y conductuales, lo que requiere acciones intersectoriales y prácticas de enfermería centradas en la continuidad de la atención y el fortalecimiento de las políticas públicas para combatir el VIH.

## INTRODUCCIÓN

La infección por el Virus de Inmunodeficiencia Humana (VIH) y el síndrome de inmunodeficiencia adquirida (sida) siguen siendo importantes problemas de salud pública mundial. Hasta 2023, más de 42,3 millones de personas habían fallecido a causa del sida, de las cuales cerca de 630 000 solo en ese año, en gran parte debido a infecciones oportunistas asociadas a la falta de adherencia a la terapia antirretroviral (TARV)^(1)^.

Con el fin de hacer frente a esta situación, el Programa Conjunto de las Naciones Unidas sobre el VIH/sida (ONUSIDA) propuso la meta “95-95-95”, alineada con el Objetivo de Desarrollo Sostenible (ODS) n.º 3, que busca eliminar la epidemia para 2030^(1)^. A pesar de los avances logrados, Brasil aún no ha alcanzado plenamente estas metas, habiendo cumplido solo el tercer hito, relacionado con la supresión viral^(2)^.

Entre 2007 y 2023, el país registró 489 594 casos de VIH, de los cuales el 19,1% se produjeron en la región Sur y 42 456 en Rio Grande do Sul^(2)^. Este estado mantiene históricamente altas tasas de incidencia y mortalidad, lo que pone de manifiesto la necesidad de fortalecer la red de atención de salud, especialmente en la articulación entre la Atención Primaria de Salud (APS) y los Servicios de Atención Especializada (SAE), fundamentales para el seguimiento longitudinal y el apoyo terapéutico continuo.

El TARV constituye el principal recurso terapéutico para el control de la infección por el VIH, reduciendo la morbilidad y la mortalidad, previniendo las hospitalizaciones e interrumpiendo la cadena de transmisión mediante el concepto “Indetectable = Intransmisible (I = I)” ^(3)^. Sin embargo, la interrupción o la adherencia irregular al tratamiento sigue representando un obstáculo significativo, influenciado por vulnerabilidades sociales, barreras de acceso, estigma y consumo de sustancias psicoactivas^(4,5,6)^.

La mayoría de los estudios sobre la adherencia al TARV se centran en personas atendidas en el ámbito ambulatorio, siendo escasas las investigaciones que abordan la realidad de los pacientes hospitalizados, grupo que presenta mayor gravedad clínica y enfrenta desafíos adicionales en la continuidad de la atención^(4,7,8)^.

Por lo tanto, este estudio tiene como objetivo identificar los factores asociados a la interrupción de la terapia antirretroviral en personas que viven con el VIH ingresadas en un hospital de tamaño mediano de Rio Grande do Sul, contribuyendo a la mejora de las prácticas asistenciales y de las políticas públicas orientadas al mantenimiento del tratamiento y a la integración entre los niveles de atención.

## MÉTODO

### Diseño del Estudio, Período y Lugar

Investigación cuantitativa, de tipo cohorte retrospectiva, que analizó historias clínicas electrónicas de personas hospitalizadas con VIH ingresadas en el período comprendido entre el 1 de enero de 2022 y el 31 de enero de 2024 y a las que se hizo un seguimiento desde el ingreso hasta el alta hospitalaria o el fallecimiento, investigando la interrupción del TARV como resultado. La descripción metodológica de esta investigación se basó en las directrices de *Strengthening the Reporting of Observational Studies in Epidemiology* (STROBE).

La investigación se llevó a cabo en una unidad de hospitalización clínica de un hospital universitario situado en el estado de Rio Grande do Sul, Brasil. La elección del escenario se debió a que la institución es un centro de referencia en la atención a personas con enfermedades infecciosas. La institución es de carácter público y filantrópico, y es un centro de referencia para 33 municipios de la macrorregión Centro-Oeste de Rio Grande do Sul. Esta macrorregión abarca una población estimada de más de 500 000 habitantes. En cuanto a la unidad donde se llevó a cabo el estudio, cuenta con 34 camas, de las cuales 15 son para hombres, 15 para mujeres y cuatro de aislamiento.

### Población, Criterios de Inclusión y Exclusión

La población estuvo compuesta por personas con diagnóstico de VIH ingresadas en la unidad de hospitalización en el período comprendido entre el 1 de enero de 2022 y el 31 de enero de 2024. Este período se estableció porque, durante los años 2020 y 2021, la unidad se convirtió en centro de referencia para la atención de personas con COVID-19. De esta manera, se buscó minimizar los efectos de la pandemia en los datos obtenidos y analizados.

Los criterios de inclusión fueron: historias clínicas electrónicas de personas mayores de 18 años, diagnosticadas con VIH e internadas durante un mínimo de 72 horas. Este tiempo se consideró con el objetivo de obtener la información mínima necesaria para el estudio. El seguimiento se realizó desde el ingreso hospitalario hasta el alta o el fallecimiento. Los criterios de exclusión fueron: historias clínicas no localizadas o que no presentaban información suficiente; historias clínicas en las que el diagnóstico de VIH fue descartado o permaneció inconcluso durante la hospitalización; y personas que iniciaron el TARV durante la hospitalización, dado que el estudio se centra en la interrupción del tratamiento antes de ese evento. No se recopilaron datos de personas reingresadas después del período del estudio.

Se consideró una muestra mínima de 188 historias clínicas, calculada en base al total de hospitalizaciones por VIH en la unidad en los últimos cinco años, adoptando una tolerancia de error del 5%, un nivel de confianza del 95% y una proporción de la característica de interés de 0,5 (50%), valor conservador elegido por no conocerse la prevalencia exacta de las interrupciones de la terapia en esta población hospitalaria.

### Recolección de Datos

La recolección de datos se llevó a cabo entre agosto de 2023 y abril de 2024, y fue realizada por el autor de la investigación y por una recolectora voluntaria, ambos previamente capacitados para minimizar cualquier sesgo en la recolección.

Para la recopilación de datos se realizó una búsqueda en la base de datos electrónica de historias clínicas de la institución, que abarcaba a todas las personas ingresadas en la unidad de hospitalización durante el período del estudio. Se identificaron 3.325 ingresos y, tras la exclusión de duplicados, se incluyeron 3.258 historias clínicas. A continuación, se procedió a la identificación de los expedientes de personas que viven con el VIH. Para esta etapa, se analizaron íntegramente los expedientes para seleccionar aquellos que cumplían con los criterios de inclusión del estudio, lo que totalizó 248 expedientes de personas que viven con el VIH. Tras la aplicación de los criterios de exclusión, se obtuvo la muestra final de 208 participantes, habiéndose excluido del estudio 40 historias clínicas (15 por corresponder a hospitalizaciones de menos de 72 horas, 14 por no presentar información suficiente para las variables estudiadas y 11 por haber iniciado el uso del TARV durante la hospitalización). El flujo de la muestra detallado también se presenta visualmente en la Figura 1.

**Figura 1:** Diagrama de flujo del número de personas elegibles para el estudio (n = 208) – Santa María, RS, Brasil, 2022–2024.

Fuente: Elaborado por los autores (2025).

### Instrumento de Recopilación de Datos y Variables

El instrumento se elaboró basándose en las recomendaciones del Protocolo Clínico y las Directrices Terapéuticas para el Manejo de la Infección por el VIH en adultos del Ministerio de Salud (MS) del año 2018 y se compuso de las variables sociodemográficas sexo (masculino/femenino), color de piel (blanco y negro/mestizo), nivel educativo (menos de ocho años de estudio y más de ocho años de estudio) y clase económica. La estratificación de la clase económica se realizó de acuerdo con la clasificación ya utilizada por la institución donde se llevó a cabo el estudio, que categoriza los ingresos mensuales familiares de los participantes de la siguiente manera: Clase A: ingresos mensuales superiores a 11 262 R$, Clase B: entre 8641 y 11 261 R$; Clase C: entre 2.005 y 8.640 reales; Clase D: entre 1.255 y 2.004 reales; Clase E: entre 0,00 y 1.254 reales. También se incluyeron la orientación sexual (homosexual, heterosexual y bisexual), el estado civil (soltero y casado) y la relación estable (sí o no). En cuanto a los factores de vulnerabilidad social y conductual, se incluyeron las siguientes variables dicotómicas (sí o no): trastorno relacionado con el consumo de tabaco, trastorno relacionado con el consumo de alcohol, personas en situación de calle, consumo de drogas y/o alcohol, y privación de libertad.

La variable clínica fue el sida (sí o no). Para la clasificación de esta variable, se adoptaron los criterios del Ministerio de Salud. Se consideró que el caso era de sida cuando había un registro explícito del diagnóstico en el historial médico o cuando cumplía con criterios de laboratorio (LT-CD4+ < 200 células/mm^3^ o carga viral >1.000 copias/mL^(3)^) y/o clínicos compatibles (empeoramiento del cuadro asociado a infecciones oportunistas). En ausencia de datos de laboratorio, prevaleció el criterio clínico y/o el registro del CIE, con el fin de reducir la pérdida de casos.

Se consideró, además, el tipo de ingreso hospitalario (electivo o de urgencia), la coinfección (tuberculosis/VIH) y la carga viral (indetectable, baja y alta). La carga viral se clasificó en: indetectable, hasta 200 copias/mL (sin riesgo de transmisión sexual); baja, entre 200 y 1000 copias/mL (riesgo casi nulo de transmisión sexual); alta, superior a 1000 copias/mL (riesgo relevante de transmisión sexual)^(2)^; y los LT-CD4+ (LT-CD4+ superior a 201 células/mm^3^ y LT-CD4+ inferior a 200 células/mm^3^). Además, se consideraron los antecedentes de hospitalizaciones previas (sí o no) y el resultado de la hospitalización (alta hospitalaria o fallecimiento). Cabe destacar que el instrumento se compartió con un comité de cinco especialistas en el tema del VIH y la epidemiología, y se evaluó y validó en cuanto a su contenido y semántica mediante el cálculo del Índice de Validez de Contenido (IVC), con el fin de mejorarlo. Los ítems con un IVC inferior a 0,80 fueron revisados o eliminados.

La muestra se estratificó en dos grupos, considerando la variable de interrupción del tratamiento, que se analizó en función del criterio de tiempo. El Ministerio de Salud define la interrupción como la ausencia de retirada de la medicación durante un período superior a 28 días después de la fecha prevista para la última dispensación, asociada al abandono del seguimiento ambulatorio^(3)^. Cabe destacar que esta variable estaba disponible en el historial del paciente, ya que el SAE del municipio comparte esta información con el hospital.

En relación con el historial terapéutico, se consideraron las siguientes variables: historial de interrupción previa del TARV (sí o no), número de esquemas del TARV (primer, segundo y tercer esquema o más), unidad de retirada de la medicación (ambulatorio o Servicio de Atención Especializada/Centro de Testeos y Asesoramiento - SAE/CTA), motivo de la búsqueda del diagnóstico (infecciones oportunistas, sintomatología o prueba rápida) y tiempo de desplazamiento hasta la unidad de recogida de la medicación (menos de 30 minutos y más de 31 minutos).

Para estructurar la variable “tiempo de desplazamiento hasta el centro de recogida de medicamentos”, se utilizó la dirección de residencia y el centro de referencia de la persona. Para estimar el tiempo, la variable se midió en la herramienta *Google Maps*, tomando en cuenta el trayecto en transporte público. Específicamente, se utilizó el tiempo promedio estimado de desplazamiento en condiciones normales de tráfico, evitando la fluctuación del tráfico real, que es difícil de estandarizar retrospectivamente.

La estratificación del tiempo de desplazamiento (≤30 minutos y ≥31 minutos) se definió considerando las características geográficas y de movilidad del municipio, en las que los desplazamientos superiores a 30 minutos representan una barrera de acceso frecuentemente observada en los trayectos urbanos. Así, el punto de corte refleja tanto la evidencia de la literatura sobre barreras geográficas al acceso a los servicios de salud como la realidad local del desplazamiento urbano^(4,7)^.

### Análisis de los Datos

Se elaboró una base de datos en una hoja de cálculo de *Excel® (Microsoft, versión 10, EE. UU.)*, con doble entrada de datos, seguida de una validación para minimizar el sesgo de medición y garantizar la fiabilidad de los datos.

Tras la validación de la hoja de cálculo y la realización de correcciones, los datos se transfirieron a una base definitiva, la cual se importó para su análisis al *software Statistical Package for the Social Sciences* (SPSS), versión 20.0.

Para el análisis de los datos, se utilizó estadística descriptiva para la caracterización de la muestra. Para las variables categóricas, se presentaron la frecuencia absoluta (n) y relativa (%). Para las variables numéricas, se verificó la normalidad de la distribución mediante la prueba de *Kolmogorov-Smirnov*. Las variables con distribución normal se describieron mediante la media y la desviación estándar (DE±), mientras que las variables con distribución asimétrica se presentaron mediante la mediana y el intervalo intercuartílico.

La asociación entre las variables categóricas se evaluó mediante las pruebas de chi-cuadrado de Pearson y la prueba exacta de Fisher, cuando era apropiado, es decir, cuando no se cumplían las premisas del chi-cuadrado, como en el caso de celdas con recuentos esperados menores de cinco, por ejemplo. Se consideró un nivel de significación estadística del 5% (p < 0,05).

También se realizó un análisis multivariante mediante regresión de Poisson con varianza robusta, con el objetivo de identificar los factores asociados a la interrupción de la terapia antirretroviral. Antes de la construcción de los modelos, se verificó la multicolinealidad entre las variables predictoras utilizando el Factor de Inflación de la Varianza (VIF), con valores aceptables por debajo de 5. Las variables se insertaron en bloques temáticos sucesivos: sociodemográfico (Bloque 1), vulnerabilidades sociales y conductuales (Bloque 2) e historial clínico/terapéutico (Bloque 3). Las variables con un valor de p < 0,20 en el análisis bivariado se consideraron para su inclusión en el modelo inicial de cada bloque, criterio recomendado por la literatura científica para maximizar la probabilidad de inclusión de variables relevantes en el modelo multivariado^(9)^. La permanencia en el modelo final se definió en función de la significación estadística (p < 0,05) y la relevancia epidemiológica.

El modelo conceptual de este estudio postula que la interrupción del TARV está influenciada por vulnerabilidades sociales, conductuales y el historial clínico/terapéutico. El resultado principal, la interrupción del TARV, se definió según los criterios del Ministerio de Salud. Para la construcción del modelo causal, se consideraron posibles variables de confusión, ampliamente evidenciadas en la literatura como factores que influyen en el resultado de la interrupción del TARV, incluyendo la carga viral, el recuento de CD4+, el diagnóstico de sida, el resultado de la hospitalización, el tipo de ingreso hospitalario, la interrupción previa y la coinfección por tuberculosis y VIH^(1,3,4,7,8)^. El impacto de estas variables de confusión se ajustó en los análisis multivariados para estimar mejor la asociación entre los factores de interés y la interrupción del TARV.

Este estudio presenta algunas limitaciones inherentes a su diseño retrospectivo. En primer lugar, la dependencia de los registros en las historias clínicas puede dar lugar a un sesgo de información (subnotificación), ya que la calidad y la exhaustividad de los datos dependen del registro clínico. En segundo lugar, el estudio se llevó a cabo en un hospital de referencia que concentra los casos más graves, lo que puede generar un sesgo de selección y limitar la generalización de los resultados a otras poblaciones de personas con VIH. Por último, el uso de datos secundarios impide establecer una causalidad definitiva.

### Aspectos Éticos

El estudio se llevó a cabo de conformidad con las directrices éticas nacionales e internacionales, y fue aprobado por el Comité de Ética en Investigación de la Universidad Franciscana bajo el dictamen n.º 5.828.312. En cuanto al Término de Consentimiento Libre e Informado, este fue dispensado, dado que los datos se obtuvieron a través de historias clínicas electrónicas.

## RESULTADOS

Se consideraron elegibles 248 historias clínicas de personas que viven con el VIH hospitalizadas durante el período del estudio. De estas, se excluyeron 40 (15 por hospitalizaciones inferiores a 72 horas, 14 por información insuficiente y 11 por inicio del TARV durante la hospitalización). Así, la muestra final estuvo compuesta por 208 historias clínicas, de las cuales 82 (39,4%) presentaban interrupción del TARV y 126 (60,6%) no.

La edad media de los participantes fue de 39,6 años (DE ± 13,2; rango 18–89), y 82 (39,4%) presentaron interrupción del TARV. El tiempo medio de interrupción fue de 14,2 meses (DE ± 8,0; mediana 12; rango 5–47 meses).

En el análisis bivariado, se observó una asociación significativa entre la interrupción del TARV y el sexo masculino, la raza negra/mestiza, un menor nivel educativo (< 8 años), la clase económica E, los ingresos individuales y la ausencia de pareja estable (p < 0,001), según la Tabla 1. Las demás variables sociodemográficas no presentaron una asociación estadísticamente significativa.

**Tabla 1:** Características sociodemográficas de las personas que viven con el VIH hospitalizadas, según la interrupción del TARV (n = 208) – Santa María, RS, Brasil, 2022–2024.

Variables	Categoría	Interrupción		Sin interrupción		Total		Valor p^* †^
		n	%	n	%	n	%	
Sexo								< 0,001^†^
	Masculino	66	80,5	58	46,0	124	59,6	
	Mujeres	16	19,5	68	54,0	84	40,4	
Color autodeclarado								< 0,001^†^
	Negra/Marrón	67	81,7	52	41,3	119	57,2	
	Blanca	15	18,3	74	58,7	89	42,8	
Nivel de escolaridad								< 0,001^†^
	0–8	66	80,5	27	21,4	93	44,7
	≥ 9	16	19,5	99	78,6	115	55,3	
Clase económica^§^								< 0,001^‡^
	Clase C	1	1,9	52	98,1	53	25,5	
	Clase D	14	22,6	48	77,4	62	29,8	
	Clase E	67	72,0	26	28,0	93	44,7	
Ingresos mensuales								< 0,001^†^
	Individual	72	87,8	41	32,5	113	54,3	
	Familiar	10	12,2	85	67,5	95	45,7	
Orientación sexual								0,441^†^
	Homosexual	27	45,8	32	54,2	59	28,4	
	Heterosexual	46	36,6	87	65,4	113	63,9	
	Bisexual	9	56,2	7	43,8	16	7,7	
Estado civil								< 0,001^†^
	Soltero/a	74	90,2	36	28,6	110	52,9	
	Casado/a	8	9,8	90	71,4	98	47,1	
Pareja estable								< 0,001^‡^
	Sí	4	4,9	90	71,4	94	45,2	
	No	78	95,1	36	28,6	114	54,8	

Leyenda: *valor p = Nivel de significación (p < 0,05);

^†^ = Prueba de chi-cuadrado de Pearson;

^‡^ = Prueba exacta de Fisher;

^§^ = Estratificación de la clase económica: realizada según la clasificación utilizada por la institución: Clase A = ingresos mensuales > 11 262 R$; Clase B = 8.641–11.261 R$; Clase C = 2.005–8.640 R$; Clase D = 1.255–2.004 R$; Clase E = 0–1.254 R$. Ningún participante fue clasificado en las clases A o B.

La interrupción del TARV se asoció de forma estadísticamente significativa con factores de vulnerabilidad social, conductual y clínica. Entre las variables conductuales, se observó una mayor prevalencia de interrupción entre las personas que consumían tabaco (86,6%), alcohol (87,8%), otras drogas (74,4%) y vivían en la calle (42,7%) (p < 0,001). No se identificó una asociación significativa con la variable privación de libertad (p = 0,378).

Entre los factores clínicos, la interrupción del TARV se asoció a la presencia de un diagnóstico de sida (86,6%), una carga viral elevada (95,1%) y un recuento de CD4+ inferior a 200 células/mm^3^ (89,0%) (p < 0,001), así como a ingresos hospitalarios de carácter urgente (81,7%). El historial de hospitalizaciones previas también mostró una asociación significativa, observándose en el 79,3% de los que interrumpieron el tratamiento (p < 0,001). En cuanto al resultado de la hospitalización, la interrupción fue más frecuente entre los pacientes que fallecieron (65,9%), en comparación con los que recibieron el alta hospitalaria, diferencia estadísticamente significativa (p < 0,001). La coinfección por tuberculosis y VIH no mostró una asociación significativa con la interrupción del TARV (p = 0,092), tal y como se expone en la Tabla 2.

**Tabla 2:** Factores de vulnerabilidad y condiciones clínicas asociadas a la interrupción del TARV en personas que viven con el VIH hospitalizadas (n = 208) – Santa María, RS, Brasil, 2022–2024.

Variables	Categoría	Interrupción		Sin interrupción		Total		Valor p^*^
		n	%	n	%	n	%	
Bloque 1 – Factores de vulnerabilidad								< 0,001^†^
Consumo de tabaco	Consume	71	86,6	40	31,7	111	53,4	
	No consume	11	13,4	86	68,3	97	46,6	
Consumo de alcohol								< 0,001^†^
	Consume	72	87,8	30	23,8	102	49,0	
	No consume	10	12,2	96	76,2	106	51,0	
Consumo de otras drogas								< 0,001^†^
	Consume	61	74,4	13	10,3	74	35,6	
	No consume	21	25,6	113	89,7	134	64,4	
Situación en la calle								< 0.001^†^
	Sí	35	42,7	10	7,9	45	21,6	
	No	47	57,3	116	92,1	163	78,4	
Privado de libertad								
	Sí	5	6,1	12	9,5	17	8,2	0,378^‡^
	No	77	93,9	114	90,5	191	91,8	
Bloque 2 – Afecciones clínicas								
Diagnóstico de sida^§^								< 0.001^†^
	Sí	71	86,6	27	21,4	98	47,1	
	No	11	13,4	99	78,6	110	52,9	
Coinfección por VIH/tuberculosis								0,092^†^
	Sí	61	74,4	20	15,9	81	38,9	
	No							
Bloque 3 – Análisis de laboratorio								
Carga viral^||^								< 0,001^‡^
	Alta	78	95,1	53	42,1	131	63,0	
	Baja	3	3,7	37	29,4	40	19,2	
	Indetectable	1	1,2	36	28,6	37	17,8	
LT CD4+^¶^								< 0,001^†^
	< 200	73	89,0	52	41,3	125	60,1	
	> 201	9	11,0	74	58,7	83	39,9	
Bloque 4 – Historial y resultado de la hospitalización								
Tipo de ingreso								< 0,001^†^
	Urgente	65	81,7	40	31,7	107	51,4	
	Optativa	15	18,3	86	68,3	101	48,6	
Historial de hospitalizaciones previas								< 0,001^†^
	Sí	65	79,3	37	29,4	102	49,0	
	No	17	20,7	89	70,6	106	51,0	
Resultado de la hospitalización								< 0,001^†^
	Alta hospitalaria	28	34,1	106	84,1	134	64,4	
	Fallecimiento	54	65,9	20	15,9	74	35,6	

Leyenda: * valor p = nivel de significación (p < 0,05);

^†^ = Prueba de chi-cuadrado de Pearson;

^‡^ = Prueba exacta de Fisher;

^§^ = El diagnóstico de sida se definió según los criterios del Ministerio de Salud: presencia de infecciones oportunistas, carga viral >1000 copias/ml, recuento de CD4+ < 200 células/mm^3^ o registro con el código CID correspondiente;

^||^ = La carga viral se clasificó como: indetectable (≤200 copias/ml), baja (200–1.000 copias/ml) y alta (>1.000 copias/ml);

^¶^ = Linfocitos T CD4+, se consideró bajo cuando era ≤ 200 células/mm^3^ y alto cuando era > 200 células/mm^3^.

En cuanto al historial terapéutico, la interrupción del TARV mostró una asociación estadística con el número de regímenes de TARV (con mayor frecuencia de interrupción en el tercer régimen o en el mayor número de regímenes realizados) y con el centro de retirada de la medicación (SAE/CTA) (Tabla 3). Además, la interrupción fue más frecuente entre las personas que buscaron el diagnóstico del VIH debido a infecciones oportunistas o sintomatología, y aquellas que tardaban más de 31 minutos en desplazarse hasta el centro de recogida de la medicación.

**Tabla 3:** Historial terapéutico entre personas que viven con el VIH hospitalizadas, según la interrupción del TARV (n = 208) – Santa María, RS, Brasil, 2022–2024.

Variables	Categoría	Interrupción		Sin interrupción		Total		Valor p^*^
		n	%	n	%	n	%	
Número de regímenes de TARV^†^								< 0,001^‡^
	Primer	7	9,2	69	90,8	76	36,5	
	Segundo	9	19,1	38	80,9	47	22,6	
	Tercero	66	77,6	19	22,4	85	40,9	
Unidad de recogida de medicamentos								< 0,001^‡^
	Ambulatorio	19	23,2	68	54,0	87	41,8	
	SAE/CTA^§^	63	76,8	58	46,0	121	58,2	
Motivo de la consulta para el diagnóstico								< 0,001^||^
	Infecciones oportunistas	3	8,6	32	91,4	35	16,8	
	Síntomas	55	61,8	34	38,2	89	42,8	
	Prueba rápida	24	28,6	60	71,4	84	40,4	
Tiempo de desplazamiento hasta el punto de recogida de la medicación^¶^								< 0,001^‡^
	≤ 30 minutos	6	7,3	104	82,5	110	52,9	
	≥ 31 minutos	76	92,7	22	15,5	98	47,1	

Leyenda: * valor p = Nivel de significación (p < 0,05);

^†^ = Terapia antirretroviral;

^‡^ = Prueba de chi-cuadrado de Pearson;

^§^ SAE/CTA = Servicio de Atención Especializada/Centro de Pruebas y Asesoramiento;

^||^ = Prueba exacta de Fisher;

^¶^ = Tiempo de desplazamiento estimado mediante Google Maps (transporte público, tráfico normal), con un umbral de corte ≤30 min y ≥31 min, definido según las características locales y las pruebas sobre las barreras geográficas de acceso.

En el análisis multivariante mediante regresión de Poisson con varianza robusta (Tabla 4), las variables que se mantuvieron como significativamente asociadas a la interrupción del TARV fueron: sexo masculino (OR = 1,58; IC del 95%: 1,42–1,71), trastorno relacionado con el consumo de alcohol (PR = 1,46; IC del 95%: 1,10–1,97), consumo de otras drogas (PR = 1,98; IC del 95%: 1,36–2,54), situación de persona sin hogar (PR = 1,38; IC del 95%: 1,12–1,78), tiempo de desplazamiento >31 minutos (RP = 1,67; IC del 95%: 1,13–1,89), antecedentes de interrupciones previas (RP = 1,52; IC del 95%: 1,27– 1,91) y diagnóstico tardío (RP = 1,30; IC del 95%: 1,09–1,67). Estos resultados indican que los factores de vulnerabilidad social y las dificultades de acceso están fuertemente asociados a la interrupción del tratamiento.

**Tabla 4:** Modelo de regresión de Poisson con varianza robusta para los factores asociados a la interrupción del TARV entre personas que viven con el VIH hospitalizadas (n = 208) – Santa María, RS, Brasil, 2022–2024.

Variables	OR^*^ bruto	IC^†^ 95%	valor p^‡^	OR ajustada	IC del 95%	valor p
Sexo						
Masculino	1,68	1,23–2,47	0,001	1,58	1,42–1,71	< 0,033
Mujeres	1,00			1,00		
Color de piel						
Negra/Marrón	2,04	1,48–4,63	0,001	1,74	0,41–1,18	0,207
Blanca	1,00			1,00		
Nivel de estudios						
0–8	3,81	2,17–3,97	0,001	1,10	0,95–1,28	0,547
≥ 9	1,00			1,00		
Renta familiar						
Individual	2,14	1,85–3,74	0,001	1,85	0,58–6,32	0,085
Familiar	1,00			1,00		
Trastorno relacionado con el consumo de alcohol						
Sí	1,58	1,54–2,01	0,001	1,46	1,10–1,97	< 0,017
No	1,00			1,00		
Consumo de drogas						
Sí	2,45	1,45–2,89	0,001	1,98	1,36–2,54	< 0,001
No	1,00			1,00		
Situación de calle						
Sí	1,68	1,25–1,98	0,001	1,38	1,12–1,78	< 0,005
No	1,00			1,00		
Tiempo de desplazamiento						
≤ 30 minutos	1,00			1,00		
≥ 31 minutos	2,35	1,51–3,45	0,001	1,67	1,13–1,89	< 0,001
Antecedentes de interrupción						
Sí	1,89	1,89–2,36	0,001	1,52	1,27–1,91	< 0,001
No	1,00			1,00		
Unidad de recogida						
Consultas externas	1,48	0,95–1,42	0,001	1,16	0,58–1,21	0,358
SAE/CTA^§^	1,00			1,00		
Motivo de la consulta para el diagnóstico						
Signos y síntomas	1,85	1,12–2,11	0,001	1,30	1,09–1,67	< 0,048
Infecciones oportunistas	3,2	1,52–3,14	0,001	0,91	0,37–1,32	0,458
Prueba rápida	1,00			1,00		

Leyenda: * RP = Razón de prevalencia;

^†^IC = Intervalo de confianza;

^‡^valor p = Nivel de significación;

^§^SAE/CTA = Servicio de Atención Especializada/Centro de Pruebas y Asesoramiento.

## DISCUSIÓN

Este estudio identificó una prevalencia del 39,4% (n = 82) de interrupción del tratamiento antirretroviral (TARV) entre las personas que viven con el VIH hospitalizadas. Se observaron resultados similares en un estudio de cohorte realizado en China (47,1%)^(10)^ y en una investigación llevada a cabo en Ribeirão Preto/SP (39,3%)^(11)^, lo que demuestra la consistencia de los hallazgos, aun en diferentes contextos geográficos e institucionales. Estos resultados refuerzan que, incluso ante los avances terapéuticos y las políticas de acceso universal, la interrupción del TARV sigue siendo un desafío significativo en el contexto hospitalario.

Además, un estudio español evidenció que la interrupción del TARV aumenta el riesgo de hospitalización (2,92 veces) y de muerte (2,15 veces), lo que refuerza la relevancia del problema^(12)^. Estos resultados pueden estar relacionados con factores como la gravedad clínica, la presencia de comorbilidades, el consumo de sustancias psicoactivas y la desarticulación de la red de atención de salud, a menudo señalados en la literatura como determinantes de la interrupción del tratamiento durante la hospitalización^(11,13)^.

El tiempo medio de interrupción identificado (14,22 meses; DP ± 8,04) también refleja la gravedad de este fenómeno. Un estudio brasileño demuestra que los períodos prolongados de interrupción se asocian con el fracaso virológico, el aumento de la carga viral y la reducción de las posibilidades de alcanzar la indetectabilidad^(14)^, lo que agrava el riesgo de hospitalizaciones y el impacto económico sobre los sistemas de salud^(15)^.

En consonancia con la literatura, el sexo masculino demostró ser un predictor independiente de interrupción del TARV (OR = 1,58; IC 95%: 1,42–1,71), resultado corroborado por estudios nacionales e internacionales^(16,17)^. La menor adherencia entre los hombres puede reflejar patrones de masculinidad que dificultan el autocuidado y el vínculo con los servicios de salud.

Aunque otras variables, como el color de la piel, el bajo nivel educativo y los bajos ingresos, mostraron asociación en el análisis bivariado, estas no mantuvieron significación en el modelo ajustado, lo que sugiere que sus efectos pueden no estar directamente relacionados con la interrupción del TARV.

La situación de vivir en la calle (OR = 1,38; IC del 95%: 1,12–1,78) se reveló como un factor de gran impacto en la interrupción del tratamiento. La falta de vivienda constituye una barrera estructural sustancial para la continuidad del TARV, y se asocia con un mayor riesgo de hospitalización y morbilidad y mortalidad en esta población^(18)^. La literatura señala que la falta de un lugar seguro para guardar los medicamentos, el acceso irregular a los servicios y la priorización de las necesidades básicas representan desafíos significativos para la adherencia^(19)^. Estos hallazgos refuerzan la necesidad de políticas intersectoriales que integren la asistencia social, la salud y la vivienda.

El estudio confirmó que los trastornos relacionados con el consumo de alcohol (OR = 1,46; IC del 95%: 1,10–1,97) y con el uso de otras sustancias psicoactivas (OR = 1,98; IC del 95%: 1,36–2,54) se asocian de manera independiente a la interrupción del TARV. Hallazgos similares en otras investigaciones^(20,21)^ refuerzan la necesidad de acciones integradas de salud mental y reducción de daños en la atención a las personas que viven con el VIH. El consumo de sustancias interfiere directamente en la rutina de medicación y en la adherencia terapéutica, además de estar asociado al estigma y a la marginación social, factores que exigen enfoques acogedores y no punitivos por parte de los equipos de salud.

Un tiempo de desplazamiento superior a 31 minutos hasta el lugar de recogida de los medicamentos (OR = 1,67; IC del 95%: 1,13–1,89) se asoció a la interrupción del TARV. Este hallazgo refuerza las barreras estructurales, como la distancia geográfica y la escasa capilaridad de los servicios de dispensación^(22)^. Es esencial optimizar la logística de distribución y ampliar la cobertura territorial de los SAE/CTA, garantizando el acceso equitativo al TARV.

Además, los antecedentes de interrupción del TARV (OR = 1,52; IC del 95%: 1,27–1,91) fueron un fuerte predictor de una nueva interrupción. La reincidencia del abandono terapéutico pone de manifiesto la necesidad de estrategias personalizadas de seguimiento, identificación temprana y apoyo tras el alta hospitalaria^(6)^.

El presente estudio identificó una asociación entre la interrupción del TARV y marcadores clínicos de gravedad, como carga viral elevada, recuento de linfocitos T CD4+ inferior a 200 células/mm^3^, ingreso hospitalario de urgencia y ocurrencia de muerte. Estos hallazgos corroboran la literatura existente, que asocia el fracaso virológico y la inmunosupresión avanzada con peores pronósticos^(3,5,8,23)^.

En cuanto a la coinfección TB/VIH, aunque se mostró prevalente, no presentó una asociación estadísticamente significativa (p = 0,092) con la interrupción del TARV. Este resultado sugiere que, aunque la coinfección agrava el cuadro clínico, otros factores, como la adherencia previa y la gravedad, pueden mediar en esta relación. Aun así, la elevada prevalencia de coinfección observada refuerza la idea de que la tuberculosis es una complicación importante en el contexto del VIH, lo que exige intensificar las estrategias de detección e integración de la atención^(24,25,26)^.

Esta discrepancia sugiere, basándose en la experiencia acumulada en la práctica clínica y en la realización de esta investigación, que el elevado número de baciloscopias de esputo positivas en el grupo con interrupción puede reflejar la gravedad de los casos no atendidos por el servicio de referencia. En estos escenarios, la interrupción del TARV puede haber dado lugar ya a una inmunosupresión avanzada, lo que aumenta la vulnerabilidad a infecciones oportunistas activas, como la tuberculosis^(25)^. Se trata de una señal de alerta sobre fallas en el rastreo, la vinculación y la permanencia de estas personas en los servicios de salud, lo que contribuye a una evolución clínica desfavorable y a la necesidad de hospitalizaciones^(26)^. La elevada prevalencia de la coinfección en este estudio refuerza la idea de que la tuberculosis es un grave problema en Brasil, lo que exige inversiones urgentes en estrategias de detección, prevención y atención integrada, sobre todo entre las personas que viven con el VIH.

La búsqueda tardía del diagnóstico del VIH (OR = 1,30; IC del 95%: 1,09–1,67) también se asoció a la interrupción del TARV, lo que refleja un menor compromiso inicial con los servicios y una mayor complejidad clínica^(27)^. Iniciar el TARV de manera temprana sigue siendo una de las medidas más efectivas para reducir la morbilidad, la mortalidad y la transmisión.

La hospitalización debe reconocerse como una oportunidad estratégica para restablecer el vínculo y la reincorporación terapéutica. El fortalecimiento de la articulación entre hospitales, APS y SAE/CTA es esencial para garantizar la continuidad de la atención. Estrategias como la búsqueda activa, el telemonitoreo, el seguimiento multiprofesional y los planes de alta individualizados han demostrado su potencial para mejorar la adherencia^(7)^. La enfermería, por su papel continuo y cercano al paciente, ocupa un lugar central en la construcción de vínculos, la identificación de riesgos y la implementación de planes de atención personalizados. La atención integral debe incluir la recuperación de la dignidad, la identidad y la autonomía de las personas que viven con el VIH, favoreciendo el autocuidado y la adherencia.

Como limitación de esta investigación, cabe destacar el uso de registros secundarios, lo que puede generar datos incompletos. Los resultados no son generalizables a toda la población brasileña, ya que el estudio se llevó a cabo en un hospital de referencia en Rio Grande do Sul, con una probable concentración de casos más graves.

Entre las contribuciones, el estudio innova al analizar los factores de interrupción del TARV en la población hospitalizada, un tema aún poco explorado, proporcionando fundamentos teóricos y operativos para la planificación de la atención de enfermería y la gestión de la adherencia terapéutica. Las futuras investigaciones deben profundizar en las barreras a la adherencia desde la perspectiva de los usuarios y evaluar el impacto de las intervenciones intersectoriales dirigidas a grupos vulnerables.

Además, los hallazgos de este estudio dialogan con el ODS 3 y con la meta 95-95-95 de ONUSIDA, al evidenciar brechas en el logro de la adherencia plena al tratamiento entre las personas que viven con el VIH hospitalizadas. El fortalecimiento de las estrategias de atención continua, interprofesional y centrada en el usuario es fundamental para alcanzar la supresión viral sostenida y reducir la mortalidad asociada al VIH.

## CONCLUSIÓN

La interrupción de la terapia antirretroviral en personas que viven con el VIH hospitalizadas se asoció con factores como el sexo masculino, el consumo de alcohol y otras sustancias psicoactivas, la situación de vivir en la calle, antecedentes previos de interrupciones, el diagnóstico tardío del VIH y un mayor tiempo de desplazamiento hasta el centro de dispensación de la medicación. Estos hallazgos refuerzan la naturaleza multifactorial de la adherencia al tratamiento y la necesidad de intervenciones integradas que consideren las dimensiones sociales, conductuales y clínicas.

Cabe destacar la importancia de fortalecer el seguimiento durante la hospitalización y garantizar la continuidad de la atención tras el alta, mediante la coordinación entre los diferentes niveles de la red de atención de salud. Aunque la coinfección TB/VIH se mostró prevalente, no presentó una asociación estadísticamente significativa con la interrupción del TARV, lo que indica la necesidad de estudios adicionales sobre su influencia en este contexto.

Los resultados contribuyen al avance de las metas 95-95-95 de ONUSIDA y al logro del ODS 3 (Salud y Bienestar), al poner de manifiesto puntos críticos que exigen políticas públicas y estrategias innovadoras orientadas a ampliar la adherencia, reducir las interrupciones y mejorar los resultados clínicos y sociales de las personas que viven con el VIH. Las futuras investigaciones deben profundizar en la comprensión de los determinantes de la adherencia y evaluar las intervenciones que promuevan una atención continua y equitativa, fortaleciendo el compromiso global para poner fin a la epidemia.

## Data Availability

El conjunto de datos que respaldan las conclusiones de este estudio está disponible previa solicitud al autor correspondiente.
